# Petrology of weathering materials developed on granites in Biou area, North-Cameroon: implication for rare-earth elements (REE) exploration in semi-arid regions

**DOI:** 10.1016/j.heliyon.2021.e08581

**Published:** 2021-12-09

**Authors:** Elisé Sababa, Lionel G. Essomba Owona, Jean Pierre Temga, Paul-Désiré Ndjigui

**Affiliations:** Department of Earth Sciences, University of Yaoundé I, P.O. Box, 812, Yaoundé, Cameroon

**Keywords:** Climate, Weathering, Geochemistry, Enrichment factor, REE exploration

## Abstract

Due to the increasing demand resulting from the use of REE in many fields of human life, a weathering profile developed on granites in the semi-arid region of Biou area (North-Cameroon) has been characterized for rare-earth elements (REE) exploration. The mineralogical compositions of weathered materials were revealed by X-ray diffraction (XRD). X-ray Fluorescence (XRF) and Inductively Coupled Plasmas-Mass Spectrometry (ICP-MS) have been used to determine the geochemical composition of granites and the overlying weathered materials. The S-type and peraluminous granites are constituted by quartz, orthoclase, microcline, plagioclase, biotite, muscovite, pyroxene and opaque minerals. Accessory minerals are probably responsible for the interesting contents in REE + Y and some trace elements (e.g., Zr, Zn, Ba, Rb). The weathering profile show from the bottom to the top: (i) saprolitic horizons; (ii) lower loose clayey horizon; (iii) iron duricrust horizon; (iv) upper loose clayey horizon; (v) and organo-mineral horizon. Some weathered rock fragments remain in the loose clayey and organo-mineral horizons. The mineralogical composition of the weathering materials is dominated by illite, muscovite and feldspar. The low weathering degree of the materials is justified by the climatic and reducing conditions. The large ion lithophiles and ferromagnesian elements (Mg, Fe, V, Cu, Co, Cs Cr, Ni, Sc and Li) which are supposed to be mobile are so much accumulated in the weathering materials. REE show very low degree of fractionation in weathering profile due probably to the lack of good drainage. The whole weathering materials shows high REE + Y contents as its parent rock. Geochemical mass balance and enrichment factor reveal that REE, especially light REE, are so much enriched in the iron duricrust horizon (ion-adsorption REE deposit type). Some heavy REE are also enriched in the lower loose clayey horizon. This first survey has revealed that the weathering materials developed on granites in Biou area are favorable for further REE exploration.

## 1 Introduction

Rare-earth elements are found in several types of deposits and can be hosted in primary or secondary materials. Most of the world's REE production (>80%) results from carbonatite-related bastnäesite and monazite deposits (Roskill, 2011). This is the case of the Bayan Obo in northern China (Fan et al., 2016; Smith et al., 2016), the Mountain Pass in USA (Castor, 2008), the Maniuping and Dalucao in Southwestern China (Liu and Hou, 2017), and the Dong Pao in Northeastern Vietnam (Verplanck et al., 2016). Rare-earth elements (REE: Lanthanum, Cerium, Praseodymium, Neodymium, Samarium, Europium, Gadolinium, Terbium, Dysprosium, Holmium, Erbium, Thulium, Ytterbium and Lutetium) can be grouped into: light REE (LREE: from Lanthanum to Europium) and heavy REE (HREE: from Gadolinium to Lutetium). Yttrium can be added to REE for their similarity in chemical properties. REE are very important for human being due to their wide range application in several domains (e.g., technologies, electronic devices, automobiles, national security applications…). As a result, their demand is always growing and exploration for potential new deposits is incited (Li et al., 2017).

Climate, relief, parent rock and time critically control weathering profile formation and development (Islam et al., 2002; Kessoum Adamou et al., 2021). With adequate climatic parameters such as temperature and precipitation, chemical alteration of parent rock is rapid leading to the formation of new minerals (secondary minerals) (Eze et al., 2021). Petrographic characteristics and mineralogical composition of a rock are used to explain the responsible parameters of the weathering of crystalline rocks and the resulting weathered landforms (e.g., Jiménez-Espinosa et al., 2007; Gong et al., 2013, 2015). Major element distribution reflects the different mineralogical transformations during weathering. The chemical weathering indices, mass balance and enrichment factor calculations are helpful to discuss the mineralogical transformations and the behavior of elements during weathering (Nyassa Ohandja et al., 2020).

Weathering-related REE deposits or weathered crust elution-deposited REE deposits mainly include residual and ion-adsorption type. These deposits are critically controlled by the type of parent rock and generally increase in the following order: sandstone < basalt < granite (Miao et al., 2007; Sanematsu et al., 2013; Mihajlovic and Rinklebe, 2018). Prolonged weathering processes in subtropical climate zones releases REE from the primary materials which are then adsorbed on the weathering products (Sanematsu and Watanabe, 2016). This type of deposits is responsible for most of the global REE resources (Yang et al., 2013) and most of ion-adsorption deposits derive from the weathering of granitic rocks (Liu et al., 2016). In fact, during weathering processes, the accumulation of clay increases from shallow to intermediate horizons as REE contents increase (Fu et al., 2019). Generally, soils resulting from weathering of granites are enriched in light REE, especially Cerium, Neodymium and Lanthanum (Da Silva et al., 2017). Light REE are mobile compared to heavy REE during weathering. As a result, HREE are leached in high weathering degree zones (Cao et al., 2016). LREE-enrichment is linked to the nature of original materials or/and due to the formation and the stability of LREE-bearers in soils (Braun et al., 1998). The recent exploration of REE in vertisols of Kaele (North Cameroon) revealed that REE are remobilized during weathering but the low contents in the weathering profiles are explained by the nature of the parent rocks which are poor in REE (Yaboki et al., 2021). Contrariwise, according to Temga et al. (2021), Cameroon seems to have important weathering-related REE deposits that are yet to be assessed. Our efforts are to investigate REE-rich weathering materials derived from granites. A weathering profile overlying granites in a semi-arid region of North Cameroon has been selected to understand its mineralogical and chemical features.

## 2 Environmental and geological settings

The Biou area belongs tothe Benue basin, North-Cameroon. The region is characterized by a Sudano-Sahelian climate (Suchel, 1987) with two unequal seasons: eight months of dry season (from October to May) and four months of rainy season (from June to September). The annual rainfall is about 800 mm with a mean annual temperature of 30 °C. The relative air humidity still very low during the long dry season (26%) and increases highly throughout the short rainy season (80%). According to the Köppen climate classification (Köppen, 1918), it is a hot semi-arid climate. The area is typically covered by woody Sudano-guinean savanah (Letouzey, 1985). The area constitutes a vast plain having altitude varying between 200 and 400 m with inselbergs (≥1000 m high). These conditions result in the development of ferruginous soils (Endoaquerts Vertisols (USDA)) with hydromorphic soils found in marshy areas (Brabant and Gavaud, 1995). The hydrographic network of the study area is dominated by seasonal rivers and streams are temporally drained, especially in the rainy seasons. This temporal regime is very strong and rains are followed by an important evacuation of water loaded with sediments of various compositions (clay, sand, gravel and organic matter) due to the intense erosion of sandy soils.

**Figure 1:**
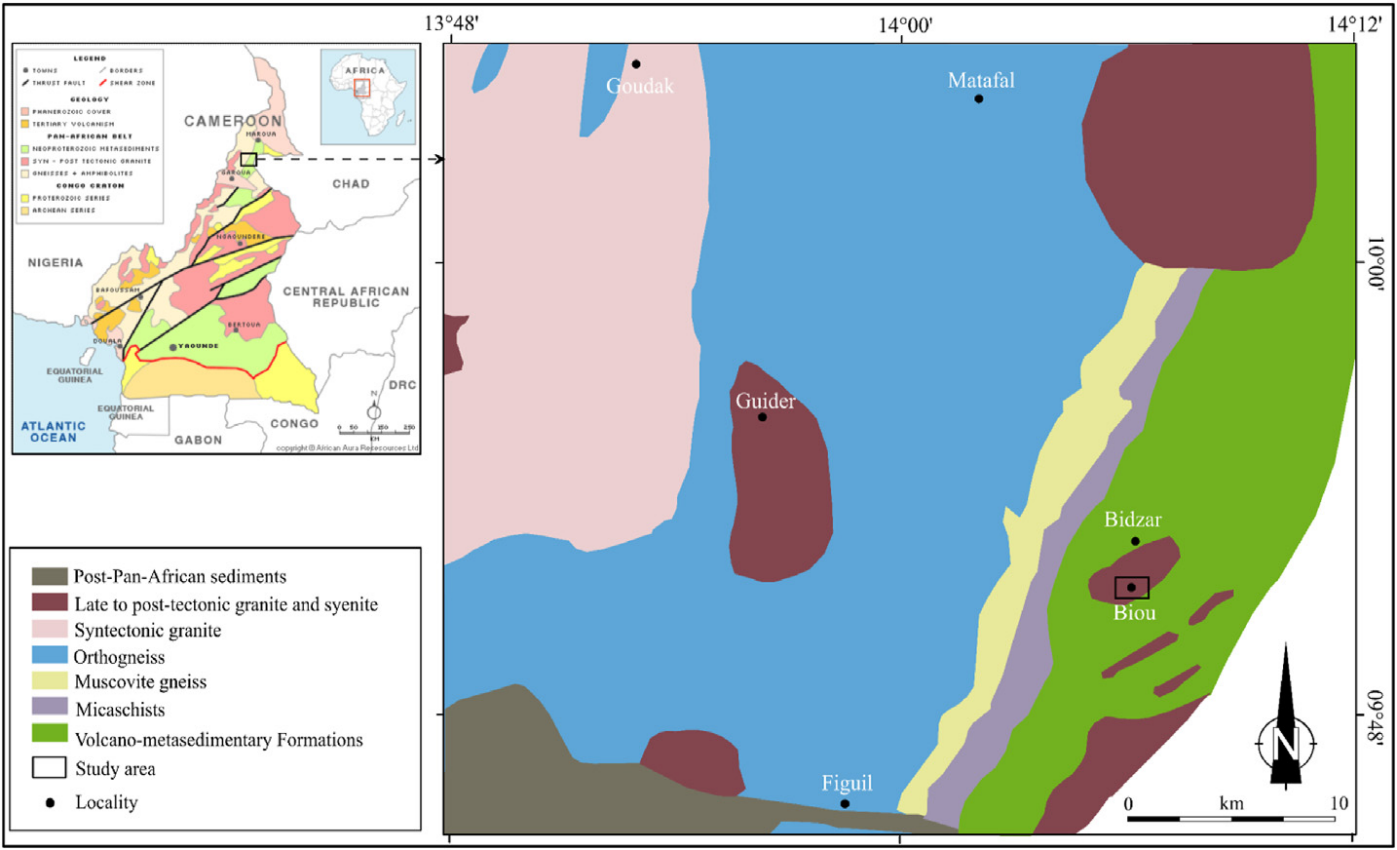
Location of the studied area in the geological map of the Biou region.

The North-Cameroon is covered by (i) Neoproterozoic (∼700 Ma) schists and gneisses of the Poli-Léré group composed by a magmatic arc (Toteu et al., 2006); (ii) Pan-African tectonic calc-alkaline granitoids (660 and 580 Ma; Penaye et al., 2006); (iii) post-tectonic alkaline granitoids as dykes cross-cut by granites and syenites; (iv) and several basins made of sediments and volcanic rocks (Toteu et al., 2004). The Pan-African basement of the study area contains distinct tonalitic orthogneisses intersected by peraluminous granite veins. The gneissic basement is made up of orthogneissitic rocks, cross-cut by pegmatite veins of magmatic composition (Guiraud et al., 1987). The geological formations in the study area are mainly metamorphic rocks (gneisses and micashists), plutonic rocks (syenite and granite) and volcano-metasedimentary (Figure 1).

## 3 Materials and methods

### 3.1 Field investigation

The weathering profile has been selected according to the lithological map and confirmed by field studies to ensure that the weathering materials are derived from granites. A reconnaissance survey campaign led to the identification of a weathering materials (located at latitude 09°52′29″N and longitude 14°06′43″E) in a stone quarry activity site. The materials were carefully scraped back to refresh and eliminate all compromising parts. Subsequently, a detailed description of the physical properties of the weathering materials was done following the FAO Guideline for soil profile description. Thereafter, hand samples were collected based on the variations in color, structure, texture and weathering assessed through hand sample observations.

### 3.2 Laboratory analysis

Thin sections of granite samples were made and microscopic observations were performed with a polarized microscope. The physico-chemical parameters of the weathering materials were determined. Ten (10) grams of powdered samples were introduced in 100 ml beakers of distilled water. The mixtures were homogenized using a spatula and a magnetic or electric stirrer for a period of 15–30 min per test. The pH data were then determined using an HACH-HQ11d brand electric pH meter calibrated to values between 0 and 14 which were coupled to the measurements of Eh. The electrochemical conductivity (EC) was measured using a HACH brand conductivity meter. The Munsell color chart was also used to determine the color of the different materials.

The bulk minerals of samples were determined by X-ray diffraction (XRD) applying the Rietveld refinement method (Ufer et al., 2008). Samples were powdered with an agate mortar and pestle. The powder sample was prepared on low background silicon disks. The analytical instrument (PAN Analytical X'PERT PRO diffractometer) was equipped with a monochromator using a Co Kα radiation of 1.7854 Å over a range of 2.5–35° 2θ and a step size of 0.05° 2θ/min at 40 kV and 45 mA. The semi-quantitative interpretation of XRD minerals was made with the High Scores Plus software. The geochemical compositions were determined after sample preparation. Samples (rocks and weathered materials) analyzed for chemical compositions were crushed using a jaw crusher with steel plates and then pulverized in a ball mill made of 99.8% Al_2_O_3_. Loss on ignition (LOI) was determined in two steps as described by Sababa et al. (2021). Powders were heated at 105 °C under nitrogen to eliminate adsorbed water and 1000 °C under oxygen to drive off all the remaining volatiles and oxidize Fe. The homogenous powdered samples were ignited and melted using a lithium tetraborate flux and analyzed with a Rigaku RIX-3000 wavelength-dispersive X-ray fluorescence (XRF) spectrometer to determine the major elements concentrations. Powder samples were prepared by acid digestion in closed beakers for the Inductively Coupled Plasmas-Mass Spectrometry (ICP-MS) analyses for trace and rare-earth element concentrations (Burnham and Schweyer, 2004). They were melted byacids (HCl and HClO_4_) at 120 °C in sealed teflon containers for one week, then in dilute nitric acid (HNO_3_) and dried. The residue was re-dissolved in the same acid mixture and dried for a second time before being dissolved in the mixture of three acids (HNO_3_, HCl and HF) at 100 °C. The solutions were then analyzed by a Perkin Elmer 5000 ICP-MS spectrometer. The instrumental precision of almost all elements was between 5 and 8.5% depending on the element concentrations. Data quality was assured by the inclusion of the international (INTL-21-37115 and INTL-21-37220), internal (IHST-21-31224 and IHST-21-31308) standards of the laboratory and duplicate samples within the analytical runs.

### 3.3 Assessment of chemical alteration

The Chemical Index of Alteration (Nesbitt and Young, 1982) was used to quantify weathering processes (Gong et al., 2013, 2015). For the application of Eq. (1), the concentrations of major elements are expressed in molar contents. CaO∗ values are the CaO molar content in silicate minerals. Subsequently, when the CaO molar content is higher than that of Na_2_O, CaO∗ is equal to the Na_2_O molar content, conversely when the CaO molar content is lower than that of Na_2_O, the CaO molar content is equivalent to CaO∗ (McLennan, 1993). The principle of CIA is based on the ratio of most mobile to least mobile elements and Al is considered as the most immobile element. Low CIA values mean low degree of weathering. Therefore, fresh rocks have the lowest CIA values; about 50% or less. CIA values close to 100% indicate a total removal of mobile elements (Ca^2+^, Na^+^ and K^+^) relative to immobile residual Al^3+^ (Nesbitt and Young, 1982; Silva et al., 2018).

(1) CIA = [Al_2_O_3_/(Al_2_O_3_ + Na_2_O + CaO∗ + K_2_O)] × 100

The Index of Lateritization is also applied in this study. Contrary to CIA, IOL is calculated using mass (wt.%) ratios of major elements (Eq. (2)). High to moderate IOL value indicates lateritization while low value (<30%) is associated with kaolinitization (Babechuk et al., 2014).

(2) IOL= (Al_2_O_3_ + Fe_2_O_3_)/(SiO_2_ + Al_2_O_3_ + Fe_2_O_3_) x 100

### 3.4 Mass balance calculation

**Figure 2:**
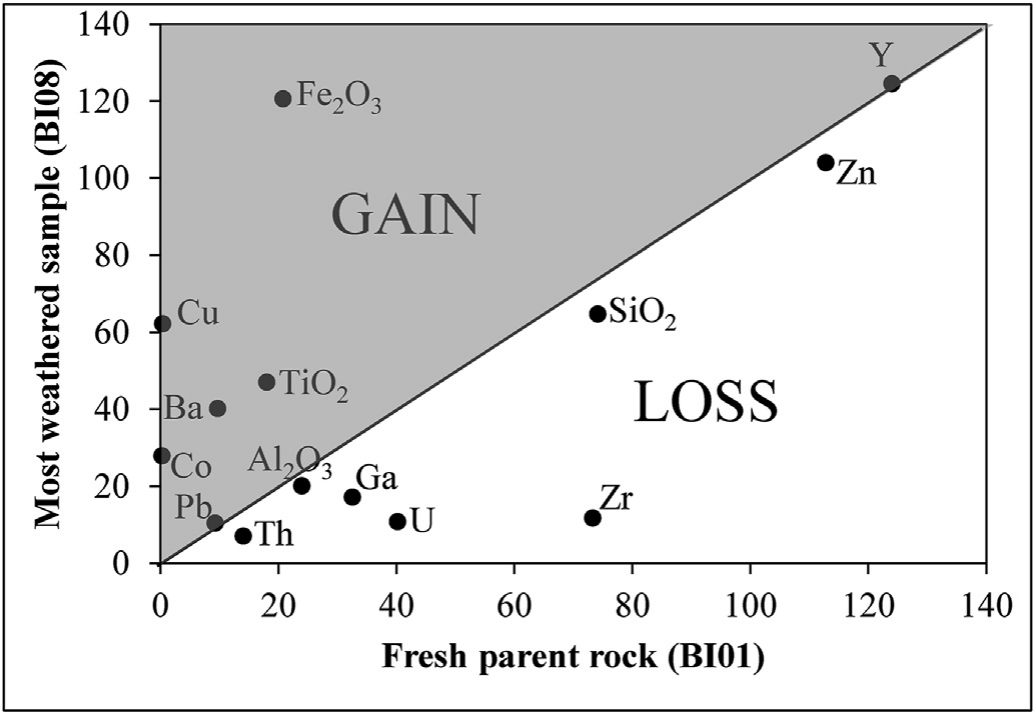
Binary diagram of Grant (most weathered sample vs. parent rock). It is performed for SiO_2_ in wt.%, Al_2_O_3_ in wt.%∗2, Fe_2_O_3_ in wt.%∗10, TiO_2_ in wt.%∗100, Cu, Co, Zn, Th in ppm, U in ppm∗10, Zr and Ba in ppm∗0.1.

The most used and reliable method of mass balance evaluation considers the element contents in different weathering materials. Mass balance expresses in percentage (%) the losses and gains. Several elements have been selected as immobile elements according to their chemical behavior in weathered products derived from an underlying parent rock to assess gains or losses during weathering (e.g., Ebah Abeng et al., 2012; Sababa et al., 2015). Gresen's equation is used to determine graphically the suitable immobile element for a study (Chu et al., 2015; Grant, 2005). Lead, Al_2_O_3_, SiO_2_, Zn and Y show positive correlation in the diagram (Figure 2) and can be reasonably used as immobile element (Chu et al., 2015). Al_2_O_3_ is a coherent oxide in the weathering materials (immobile element) and was used for mass balance calculation in this study. The losses or gains of element during weathering are calculated by Eq. (3) (Anderson et al., 2002):

(3) Percent change = 100 x [(C_j,w_/C_j,p_)/(C_Al,w_/C_Al,p_) – 1]

C_j,w_ and C_j,p_ are the contents of element in the weathered material and the parent rock, respectively. C_Al,w_ and C_Al,p_ are the contents of Al in the weathered material and the parent rock, respectively. Positive percent change reveals a gain and negative one for a loss of the element.

### 3.5 Enrichment factor calculation

The Enrichment Factor (EF) of REE in the weathered materials developed on granites was calculated. It is used to examine the surface processes which influence the distribution of chemical elements in weathering materials (Hu et al., 2013). Aluminum is selected to calculate the losses or gains of rare-earth elements for it shows homogeneous distribution in weathering materials (Nesbitt and Markovics, 1997; Andrade et al., 2019), especially in these weathered materials that were assessed. EF values are given by Eq. (4) (Blaser et al., 2000):

(4) EF = [(E/Al) _sample (weathered material)_] / [(E/Al) _background (fresh rock)_]

where Al = content of Al; E = content of the element assessed; EF value lower than unity means depletion, and EF value higher than unity expresses enrichment.

## 4 Results

### 4.1 Petrology of granites

#### 4.1.1 Petrography

The granites are massive and seem fresh. They present fine grained structure and are very hard to break. Quartz and feldspars are pinkish and locally associated with biotite which is dark.

**Figure 3:**
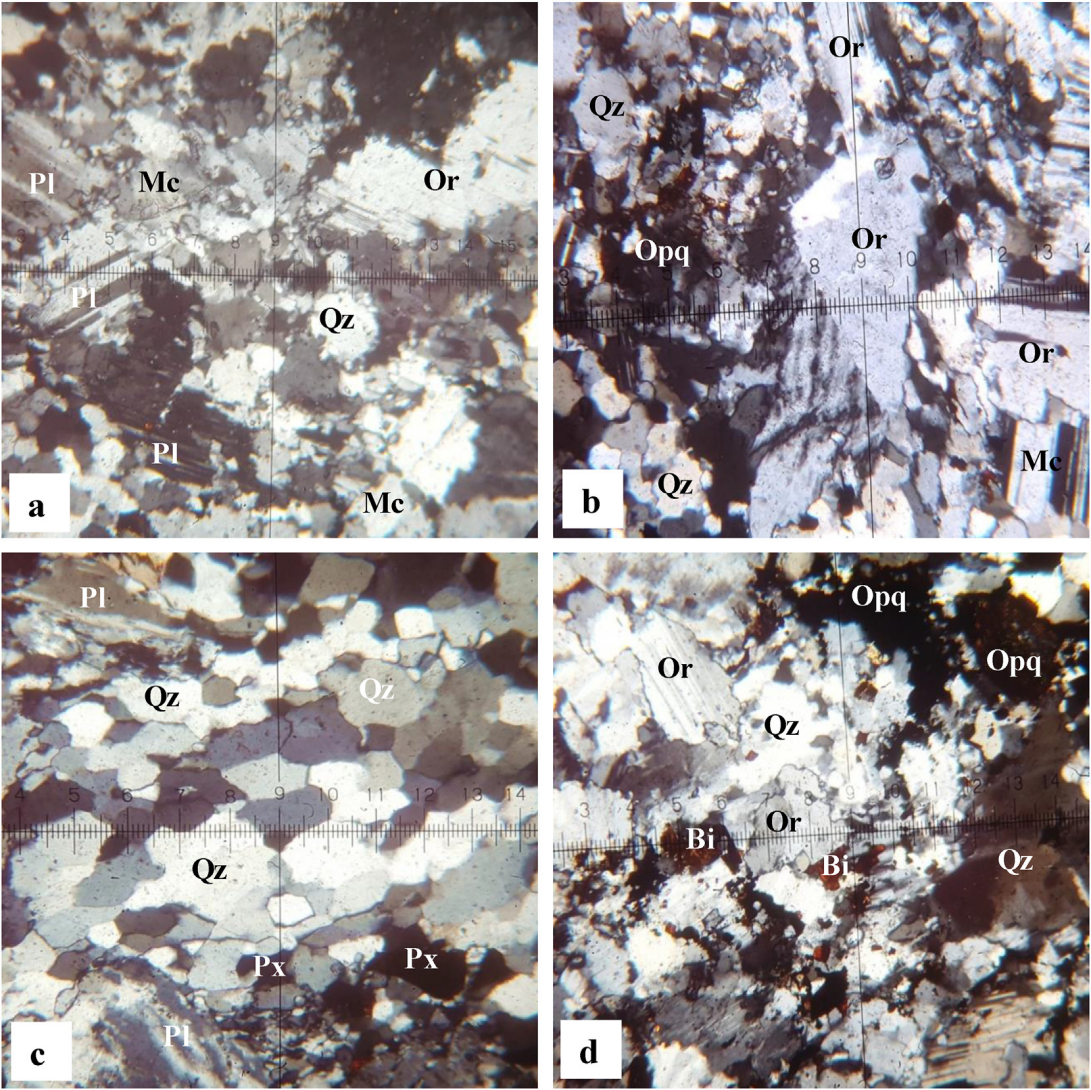
Micrographs of granites from Biou area: (a) abundance of quartz and feldspar, (b) solid state deformation of quartz and orthoclase, (c) aggregate of quartz in granular form and (d) distribution of the opaque minerals in the granites.

The granites show a granular texture (Figure 3) with amodal composition of quartz (20–25 vol.%), orthoclase (20–25 vol.%), microcline (15–20 vol.%), plagioclase (10–15 vol.%), biotite (∼10 vol.%), muscovite (∼5 vol.%), pyroxene (∼5 vol.%) and opaque minerals (∼5%). Most of those minerals occur as sub-automorphic to xenomorphic crystals. Quartz grains vary between 0.1 and 2 mm in size. They show an elongation in a preferential direction thus indicating that the rocks have undergone deformation. The elongated grains may also indicate incipient granite metamorphism. Quartz grains are sometimes found as inclusions in the orthoclase and microcline (Figure 3a). Orthoclase crystals usually occur as phenocrysts showing ex-solutions and quartz inclusions, and vary between 0.1 and 3 mm in size. Some phenocrysts are undergoing fragmentation (Figure 3b). Microcline is abundant as quartz and orthoclase with a size that varies between 0.1 and 3 mm. It presents inclusions of biotite being transformed into opaque minerals. Some crystals show inclusions of quartz and biotite as well as perthitic ex-solutions. Plagioclase does not exceed 1 mm in size. Plagioclase crystals are in frequent association with quartz, orthoclase, microcline and biotite (Figure 3a). Biotite is present in granites of Biou as elongated lamellae and similar to plagioclase in size. It is very scattered in the rock and seems to mold other minerals. It is sometimes included in the orthoclase or in the process of destabilization into opaque minerals. Muscovite occurs as thin elongated rods and like biotite, it seems to mold other minerals. Pyroxene occurs in a fine size (<0.2 mm) and some crystals show destabilization into opaque minerals. These opaque minerals derived from the alteration of biotite or pyroxene (Figure 3c). They appear in xenomorphic form (Figure 3d). The transformation most often takes place from the edge to the core of the mineral.

#### 4.1.2 Geochemistry

**Figure 4:**
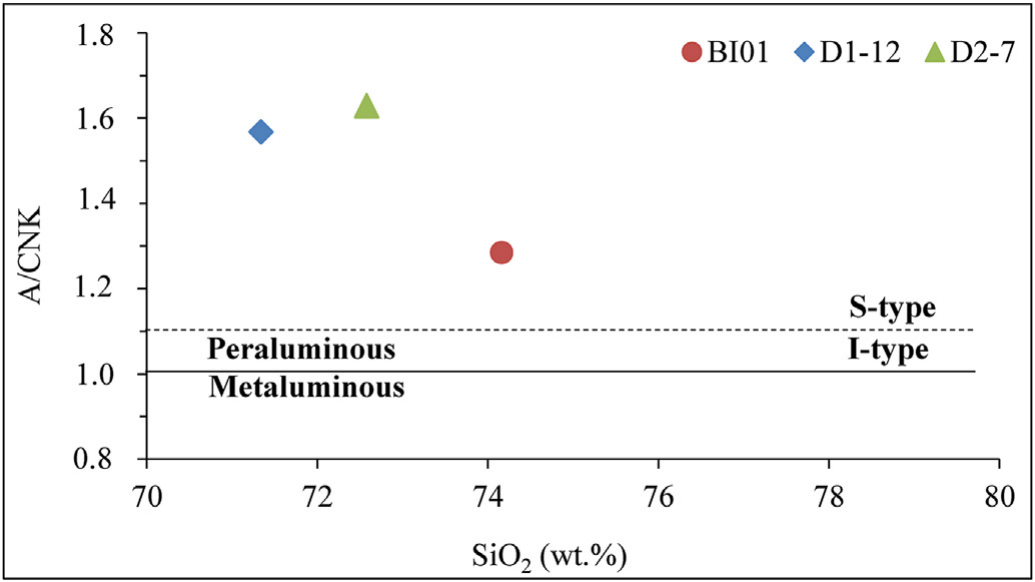
A/CNK (Al_2_O_3_/CaO + Na_2_O + K_2_O) versus SiO_2_ plot (Chappell and White (1974) portraying the S-type composition of granites (D1-12 and D2-7: data from Fu et al. (2019)).

**Figure 5:**
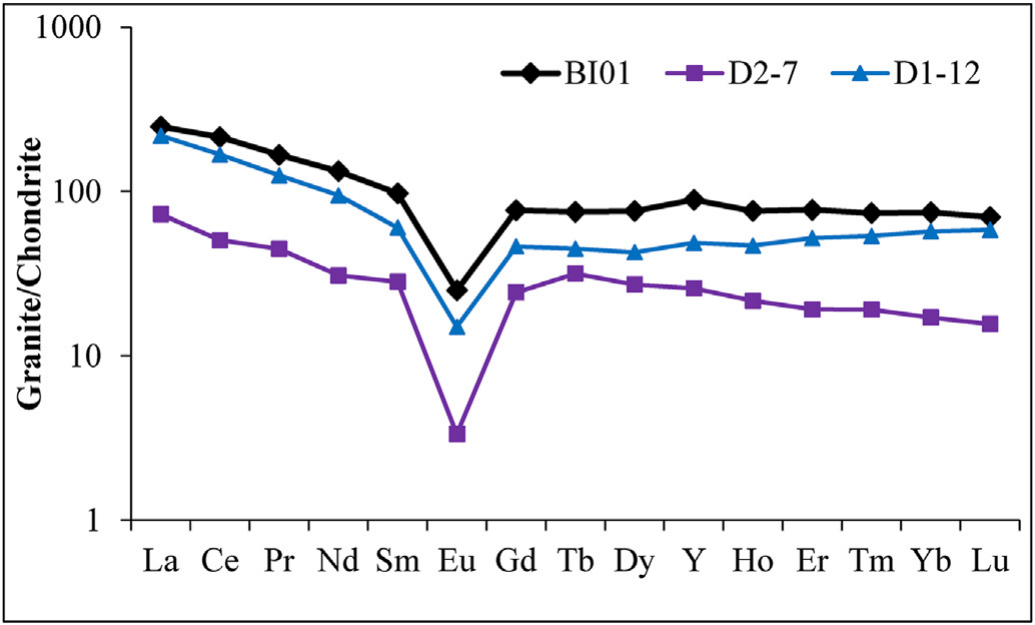
Chondrite-normalized REE (Pourmand et al., 2012) patterns for S-type granites (D1-12 and D2-7: data from Fu et al. (2019)).

The SiO_2_ content is 74.16 wt.% and Al_2_O_3_ is 11.97 wt.% in the parent rock (Table 1). The granites are characterized by an alkali enrichment (Na_2_O = 4.15 wt.%; K_2_O = 4.46 wt.%) and a depletion of ferromagnesian major elements (Fe_2_O_3_ (2.76 wt.%), MgO (0.11 wt.%), MnO (0.06 wt.%) and TiO_2_ (0.18 wt.%)). SiO_2_/Al_2_O_3_ and Na_2_O/K_2_O ratios confirm the dominance of SiO_2_, Al_2_O_3_ and to a lesser extent K_2_O over the other major elements (Table 1). The LOI (<1 wt.%), CIA (50.63 %), IOL (16.57 %) values (Table 1) show that the granite (parent rock) is still fresh (Nesbitt and Young, 1982; Babechuk et al., 2014; Silva et al., 2018) as expected. The binary diagram of A/CNK (Al_2_O_3_/CaO + Na_2_O + K_2_O) versus SiO_2_ for granite sample (Figure 4; Chappell and White, 1974) show that the rock is peraluminous and belongs to S-type granites (A/CNK> 1.1). As for ferromagnesian major elements, the granites possess low contents in Sc, V, Cr, Co and Ni (ferromagnesian trace elements) (Table 2). Otherwise, they have significant concentrations in Zr (733 ppm), Y (124.06 ppm), Zn (112.80 ppm), Ba (97.20 ppm), Rb (82.85 ppm) and Nb (65.93 ppm) (Table 2). The rare-earth element concentration in the granites is significant (ƩREE = 366 ppm; Table 3). The LREE/HREE ratio (4.11) reveals a high content in light REE compared to heavy REE (Table 3). The normalization to chondrite according to Pourmand et al. (2012) reveals REE + Y enrichments in granites relative to chondrite data (Figure 5). The spectra indicate (Figure 5; Table 3): (i) LREE enrichment relative to HREE; (ii) the presence of very pronounced negative Eu anomaly (Eu/Eu∗ = 0.29); (iii) and very low value of the (La/Yb)_N_ ratio expressing low fractionation degree.

**Table 1:** Distribution of major elements (wt.%) in weathering profile from Biou area.

	d.l.	Parent rock	Coarse saprolite	Fine saprolite	Lower loose clayey horizon			Iron duricrust horizon	Upper loose clayey horizon	Organo-mineral horizon
		BI01	BI02	BI03	BI04	BI05	BI07	BI08	BI09	BI06
SiO_2_	0.04	74.16	73.75	73.51	79.07	85.38	82.72	64.67	84.98	75.69
Al_2_O_3_	0.02	11.97	12.86	13.73	9.54	6.86	7.26	10.05	6.76	10.93
Fe_2_O_3_	0.01	2.76	2.76	3.27	2.54	2.13	2.99	12.59	1.74	2.82
MnO	0.002	0.06	0.01	0.03	0.02	0.02	0.02	0.47	0.01	0.04
MgO	0.01	0.11	0.28	0.31	0.16	0.16	0.22	1.08	0.12	0.20
CaO	0.006	0.71	0.70	0.07	0.03	0.02	0.10	0.42	0.03	1.13
Na_2_O	0.02	4.15	3.23	1.47	1.41	0.31	0.33	0.10	0.68	2.14
K_2_O	0.01	4.46	4.66	4.43	3.58	2.33	1.65	0.82	2.73	4.00
TiO_2_	0.01	0.18	0.21	0.21	0.16	0.17	0.22	0.47	0.14	0.27
P_2_O_5_	0.002	0.01	0.01	0.01	0.01	0.01	0.06	0.04	0.01	0.02
LOI	-	0.70	1.37	2.39	2.22	2.30	3.47	8.37	1.62	2.98
Total	-	99.27	99.84	99.43	98.74	99.69	99.04	99.08	98.82	100.22
SiO_2_/Al_2_O_3_	-	6.20	5.73	5.35	8.29	12.45	11.39	6.43	12.57	6.92
CIA	-	50.63	55.35	65.50	60.56	69.22	74.86	89.99	62.29	58.09
IOL	-	16.57	17.48	18.78	13.25	9.53	11.03	25.93	9.09	15.37

d.l.: detection limit.

LOI: loss on ignition.

CIA = [Al_2_O_3_/(Al_2_O_3_ + CaO∗ + Na_2_O + K_2_O)] x 100.

IOL = [(Al_2_O_3_ + Fe_2_O_3_)/(SiO_2_ + Al_2_O_3_ + Fe_2_O_3_)] x 100.

**Table 2:** Distribution of trace elements (ppm) in weathering profile from Biou area.

	dl	Parent rock	Coarse saprolite	Fine saprolite	Lower loose clayey horizon			Iron duricrust horizon	Upper loose clayey horizon	Organo-mineral horizon
		BI01	BI02	BI03	BI04	BI05	BI07	BI08	BI09	BI06
Cr	2.90	<dl	<dl	<dl	20.00	21.00	27.00	35.00	16.00	10.00
V	0.40	0.50	2.00	5.20	16.40	13.70	24.30	72.30	11.20	13.20
Ni	0.60	<dl	1.40	1.40	6.00	7.70	17.20	32.50	10.60	6.90
Co	0.09	0.23	0.43	1.67	1.91	1.53	4.82	27.80	1.38	3.03
Zn	2.00	112.80	142.40	137.00	86.40	70.40	68.00	104.00	57.60	75.30
Sc	0.17	<dl	0.50	0.50	1.50	1.30	1.90	9.30	1.10	1.30
Cu	0.40	0.40	1.10	2.10	4.30	2.20	7.60	62.20	4.20	2.60
Ba	1.30	97.20	101.50	139.10	68.00	53.30	43.60	400.10	49.10	112.20
Pb	0.29	9.25	5.90	23.50	22.29	14.25	14.90	10.38	9.16	15.32
Y	0.09	124.06	110.81	104.33	78.56	68.97	37.07	124.47	56.12	96.28
Ga	0.04	32.52	37.46	38.90	26.92	17.93	21.44	17.14	17.74	28.98
Th	0.027	14.03	10.64	11.76	9.52	8.51	7.78	7.06	8.84	11.02
U	0.01	4.18	2.42	2.08	2.69	2.45	1.58	1.78	2.28	2.50
Zr	4.00	733.00	732.00	727.00	743.00	671.00	489.00	117.00	646.00	840.00
Li	0.24	<dl	10.00	5.90	6.30	6.50	7.10	10.80	4.50	8.80
Sb	0.025	0.30	0.34	0.11	0.17	0.15	0.33	0.32	0.15	0.20
Nb	0.05	65.93	70.75	84.66	54.57	59.49	54.66	13.87	56.6	63.59
Hf	0.09	17.85	15.14	16.66	16.04	16.26	11.45	3.14	14.18	18.51
Be	0.024	8.29	5.23	6.97	2.83	1.91	2.10	4.73	1.64	2.92
Cd	0.018	0.25	0.05	0.04	0.03	0.03	0.02	0.05	0.05	0.04
Mo	0.08	0.55	0.23	1.40	1.02	1.03	0.84	4.49	1.05	0.77
Sn	0.17	7.86	7.73	7.36	5.20	4.37	6.55	2.42	2.76	4.87
W	0.023	1.12	1.29	3.93	1.29	1.08	1.16	2.32	1.08	2.04
Cs	0.006	0.123	0.45	0.48	0.38	0.38	0.59	1.08	0.33	0.42
Rb	0.15	82.85	123.35	131.25	75.54	59.28	60.75	51.57	63.71	90.18
Sr	1.30	10.60	7.10	4.80	4.80	4.30	8.70	25.60	5.80	16.40
Ta	0.015	4.75	3.36	4.76	3.88	4.42	3.77	0.87	4.23	4.15
Bi	0.05	0.15	0.10	0.15	0.22	0.43	0.37	0.23	0.79	0.17

dl: detection limit.

**Table 3:** Distribution of trace earth elements (ppm) in weathering profile from Biou area.

	dl	Parent rock	Coarse saprolite	Fine saprolite	Lower loose clayey horizon			Iron duricrust horizon	Upper loose clayey horizon	Organo-mineral horizon
		BI01	BI02	BI03	BI04	BI05	BI07	BI08	BI09	BI06
La	0.09	61.30	54.30	58.70	11.60	6.50	14.10	148.40	5.00	17.20
Ce	0.17	135.58	132.93	168.81	133.21	63.26	55.57	114.39	25.49	72.95
Pr	0.019	16.02	12.64	12.24	2.62	1.60	3.60	41.72	1.31	4.48
Nd	0.11	64.54	48.88	47.90	10.04	6.62	14.13	171.58	5.48	17.60
Sm	0.05	15.16	9.71	7.64	2.37	1.71	3.02	34.60	1.72	4.44
Eu	0.008	1.50	0.99	0.84	0.3	0.27	0.33	3.82	0.21	0.65
Gd	0.04	16.08	11.29	8.91	3.69	3.39	3.14	30.77	3.15	7.89
Tb	0.009	2.84	2.36	1.89	1.04	0.96	0.67	3.85	0.84	1.86
Dy	0.04	19.59	17.81	14.57	9.52	9.04	5.31	21.48	7.21	14.08
Ho	4.21	3.79	3.79	3.24	2.34	2.27	1.27	3.93	1.74	3.23
Er	0.04	12.91	11.72	10.37	8.16	7.92	4.54	10.45	6.04	10.36
Tm	0.005	1.93	1.74	1.56	1.28	1.26	0.75	1.34	0.98	1.6
Yb	0.008	12.65	11.65	10.39	8.74	8.57	5.59	7.81	6.67	11.03
Lu	0.005	1.79	1.72	1.48	1.23	1.21	0.85	1.04	0.94	1.64
REE	-	365.68	321.53	348.54	196.14	114.58	112.87	595.18	66.78	169.01
LREE	-	294.10	259.45	296.13	160.14	79.96	90.75	514.51	39.21	117.32
HREE	-	71.58	62.08	52.41	36.00	34.62	22.12	80.67	27.57	51.69
LREE/HREE	-	4.11	4.18	5.65	4.45	2.31	4.10	6.38	1.42	2.27
Ce/Ce∗	-	1.05	1.17	1.46	5.58	4.53	1.80	0.34	2.30	1.92
Eu/Eu∗	-	0.29	0.98	1.06	1.06	1.17	1.12	1.22	0.94	1.14
(La/Yb)_N_	-	3.32	0.96	1.17	0.27	0.16	0.52	3.92	0.15	0.32

dl: detection limit.

LOI: Loss on ignition.

CIA = [Al_2_O_3_/(Al_2_O_3_ + Na_2_O + CaO∗ + K_2_O)] × 100).

Ce/Ce∗ = (Ce_sample_/Ce_granite_)/(La_sample_/La_granite_)^1/2^(Pr_sample_/Pr_granite_)^1/2^_._

Eu/Eu∗ = (Eu_sample_/Eu_granite_)/(Sm_sample_/Sm_granite_)^1/2^(Gd_sample_/Gd_granite_)^1/2^.

(La/Yb)_N_ = (La_sample_/La_granite_)/(Yb_sample_/Yb_granite_).

Parent rock (BI01) is normalized relative to chondrite data from Pourmand et al. (2012).

### 4.2 Petrology of weathering materials

#### 4.2.1 Morphology, physic-chemical and mineralogical features

**Figure 6:**
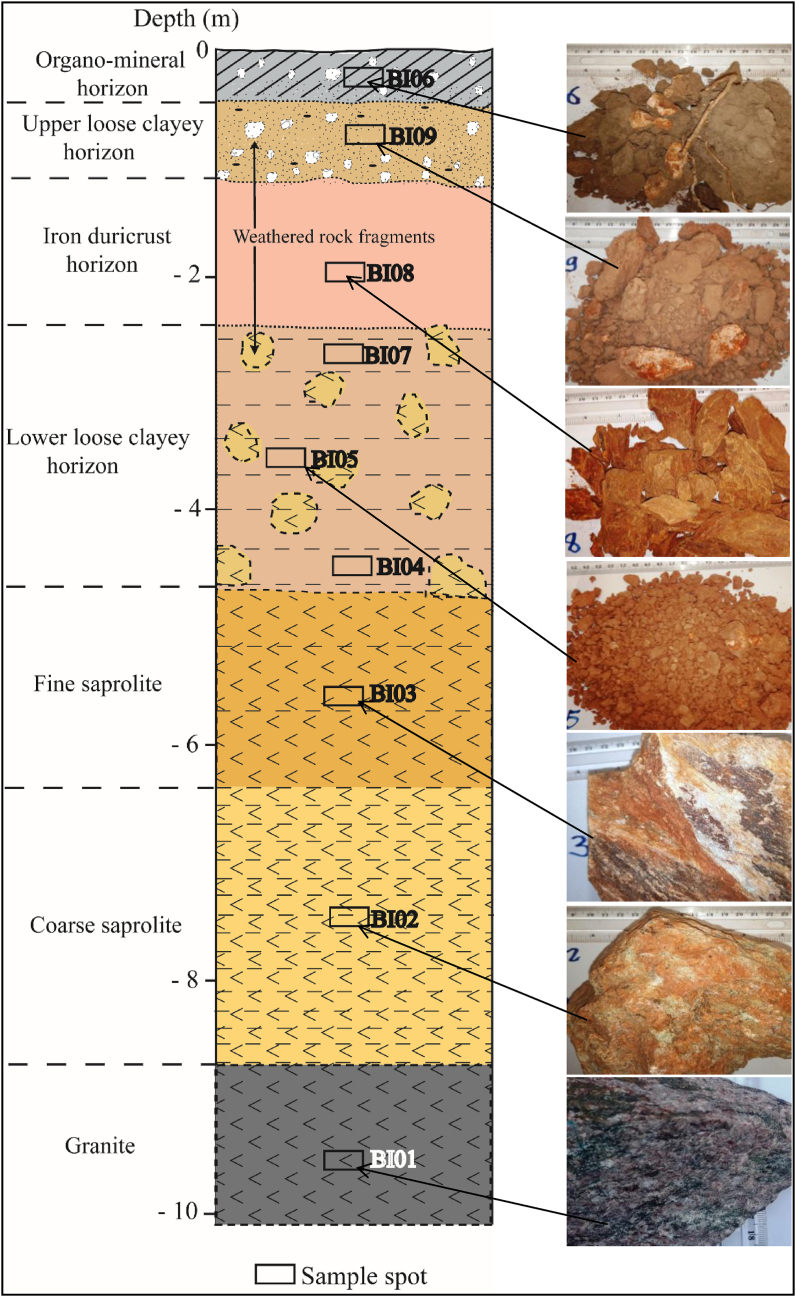
Morphological organisation of the Biou weathering profile with hand samples.

**Figure 7:**
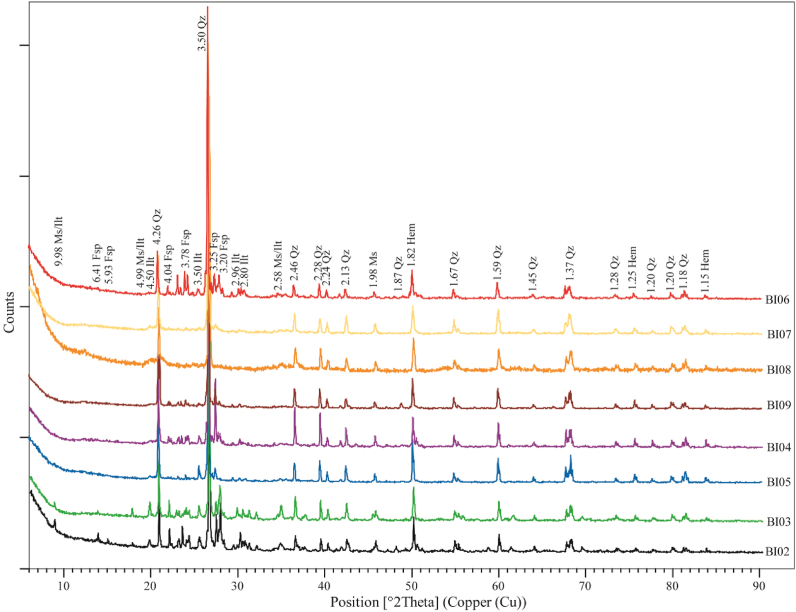
X-ray diffraction patterns of weathering materials of Biou area.

Detailed description of the morphological and physic-chemical properties of the weathering profile (Figure 6) from a semi-arid area of North Cameroon is presented in Table 4 and the mineralogical composition is shown in Figure 7.

**Table 4:** Morphological and physic-chemical properties of weathering profile from Biou area.

	Coarse saprolite	Fine saprolite	Lower loose clayey horizon			Iron duricrust horizon	Upper loose clayey horizon	Organo-mineral horizon
	BI02	BI03	BI04	BI05	BI07	BI08	BI09	BI06
Lower depth (m)	-8.70	-640	-4.90	-3.90	-3.10	-2.40	-1.30	-0.40
Munsell color (dry)	Light greenish gray GLEY2 8/1	Pinkish white 5YR 8/2	Yellowish red 5YR 5/8	Light red 2.5YR 7/6	Red 2.5YR 5/8	Light red 2.5YR 6/8	Pink 2.5YR 8/4	Gray 5YR 5/1
Rock fagment (vol%)	-	-	15	15	10	0	25	20
Structure	Massive	Massive	Granular	Granular	Massive	Massive	Granular	Granular
Consistence (dry)	Firm	Firm	Friable	Friable	Friable	Firm	Friable	Friable
Fine roots	None	None	None	None	None	None	Very few	Many
Texture (matrix)	-	-	Sandy clayey	Sandy clayey	Sandy clayey	Clayey silty	Sandy clayey	Silty clayey
pH	7.33	7.10	6.38	5.76	6.53	6.52	6.58	6.49
Eh (mV)	93.70	5.90	83.40	70.00	4.30	28.60	93.40	29.20
EC (dS/cm)	162.50	70.10	44.70	48.10	93.40	132.20	56.00	195.00

The weathering materials occur on a gross slope at 419 m of altitude. It possesses a depth of 8.5 m and from bottom to top comprising: fresh granites, coarse saprolite, fine saprolite, lower loose cleyey horizon (C horizon), iron duricrust horizon (Bt horizon), upper loose clayey horizon and organo-mineral horizon (A horizon) (Figure 7). The saprolitic horizons are made up of weathered granites showing similar structure with the parent material and low degree of weathering. The loose clayey and organo-mineral horizons comprise weathered rock fragments that never exceed 8 cm in size, making from 5 to 20% (Table 4) by volume embedded in a sandy clayey matrix. The iron duricrust horizon (Bt horizon of a Planosol) constitutes massive blocks easy to break by a harmer and show clayey silty texture. The organo-mineral horizon contains a huge amount of weathered rock fragments and fine roots (Table 4). It is a dry soil (aridisol) which is characterized by low concentration of organic matter and water deficiency.

The weathering materials developed on granites from North Cameroon are moderately acid to basic (pH = 5.76–7.33; Table 4). The lowest pH values are obtained in the lower loose clayey horizon. The pH values consistently increased down the sequence with the saprolitic levels slightly basic (Table 4). The electrode potential (Eh) shows variable behavior (4.30–93.70 mV) with the highest values in the upper loose clayey horizon and coarse saprolite. The lowest Eh values are registered in the upper part of the lower loose clayey horizon and fine saprolite. Electrical conductivity (EC) varies consistently from 44.70 dS/cm at the base of lower loose clayey horizon to 195 dS/cm in organo-mineral horizon (Table 4). The EC levels classify the weathering materials as strongly saline materials. Their mineralogical assemblage includes more primary minerals (quartz, feldspars and muscovite) than secondary minerals (illite and hematite) (Figure 7). Illite is mostly abundant at the bottom of the profile. This mineralogical composition reveals that the local environmental conditions are not favorable for hydrolysis processes.

#### 4.2.2 Geochemical characterization

SiO_2_ (64–85 wt%)is the most dominant major element followed by Al_2_O_3_ (7–13 wt%) (Table 1). The behaviors of Si and Al are close to those of the parent material. In detail, the loose clayey and organo-mineral horizons have the highest SiO_2_ contents more than the parent rock values. The lowest SiO_2_ value is registered in the iron duricrust horizon. Fe_2_O_3_ has high content in the iron duricrust horizon (12.59 wt.%) while the other horizons have low contents (<4 wt.%). Loss on ignition (LOI), a predictor of H_2_O and CO_2_ abundance (Sababa et al., 2021) mostly resulting from weathering, has higher values in the whole weathering materials than the parent rock. The LOI values reach 8.37 wt.% in the iron duricrust horizon and are from 1.37 to 3.47 in the other horizons (Table 1). The SiO_2_/Al_2_O_3_ ratios range from 5 to 13 with the maximum values in the loose clayey horizons. The Index of Lateritization (IOL) ranges from 9 to 26 % and CIA ranges between 55 and 90 %. The iron duricrust horizon has the highest IOL and CIA values (Table 1). This is due to clay accumulation in this horizon and should not be related to a higher degree of weathering.

**Figure 8:**
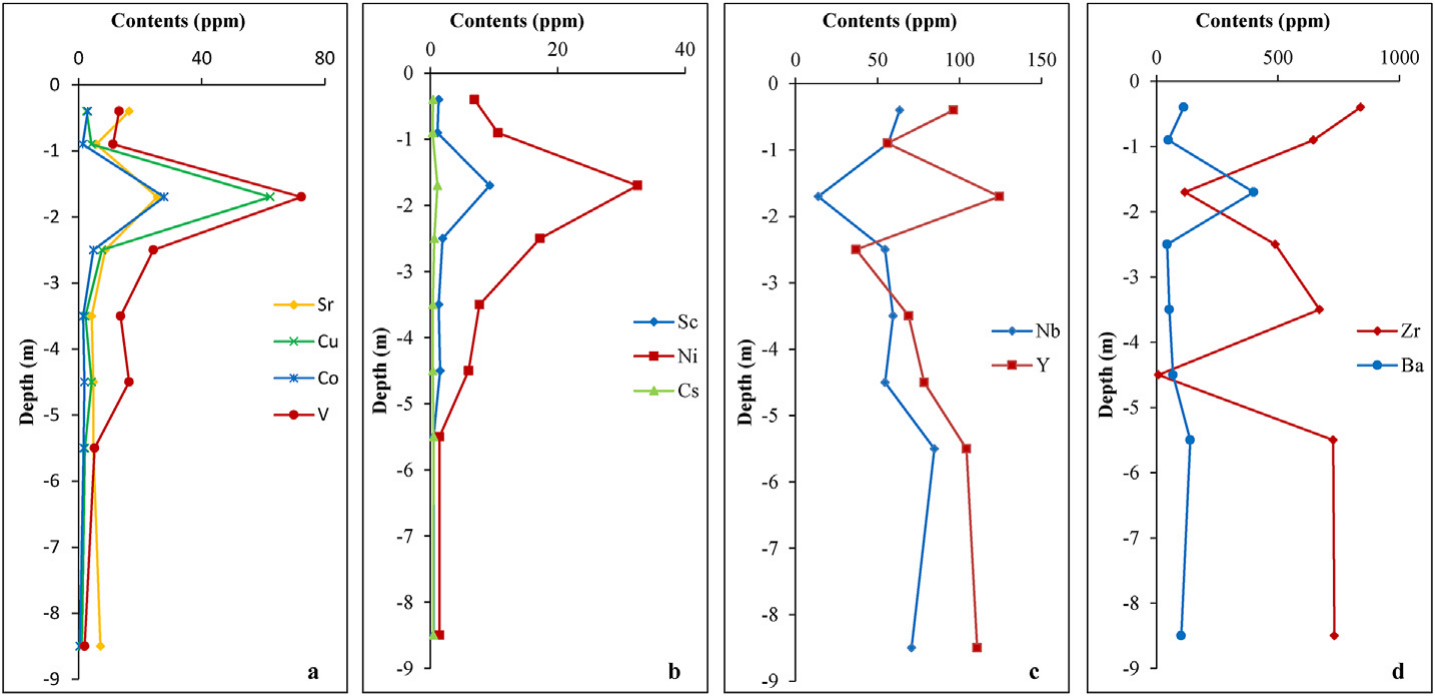
Variation of selected trace elements in the Biou weathering profile.

Amount trace elements, Zr show particularly high contents (Table 2). A part from Ba and Rb, the large ion lithophiles elements (LILE) which are considered as mobile elements, have low contents in the weathering materials excluding the iron duricrust horizon. Except Rb (51.57 ppm), all the LILE (Ba = 400.10 ppm; V = 72.30 ppm; Cu = 62.20 ppm; Co = 27.80 ppm; Sr = 25.60 ppm and Cs = 1.08 ppm) have their highest contents in the iron duricrust horizon (Figure 8a and b; Table 2). They follow a decreasing order of Ba (43–139 ppm), Rb (51–131 ppm), V (2–24 ppm), Sr (4–16 ppm), Cu (1–8 ppm), Co (0.43–5 ppm) and Cs (<1 ppm) in the other horizons. Amount the high field strength elements (HFSE: Y, Th, U, Zr, Nb, Hf, Mo, W and Ta) (largely immobile nature), only Zr (117–840 ppm), Y (37–124 ppm) and Nb (13–71 ppm) have relative high concentrations (Table 2). Low contents in Zr and Nb are surprisingly encountered in the iron duricrust horizon (Figure 8c and d). Some other trace elements like Zn (68–142 ppm) and to a lesser extent Ga (17–39 ppm) and Pb (5–22 ppm) possess significant contents in the whole weathering profile (Table 2).

**Figure 9:**
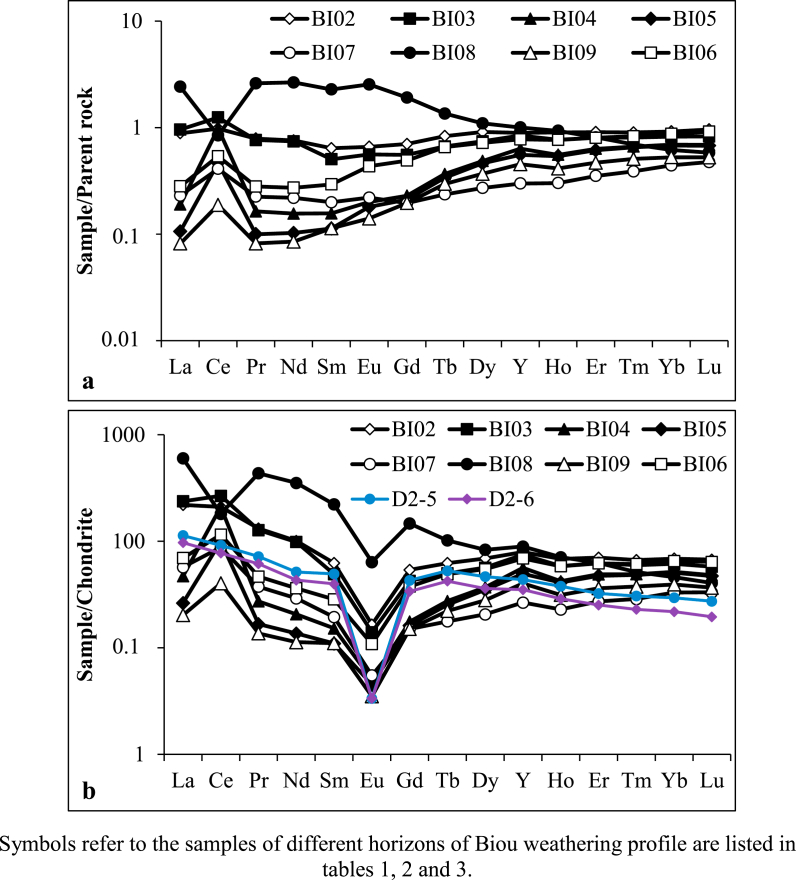
Granite- and chondrite-normalized REE patterns of weathering materials (D2-5 and D2-6: samples of semi-weathered horizon from Fu et al. (2019)).

Rare-earth element concentrations are variable along the weathering profile developed on granites of Biou (REE = 66–595 ppm). The lowest content is obtained in the upper loose clayey horizon and the peak value in the iron duricrust horizon (Table 3). As for parent rock, LREE have high contents compared to HREE (LREE/HREE = 1.42–6.38). The LREE abundance compared to HREE is low towards the surface of the profile in upper loose clayey and organo-mineral horizons. The REE + Y normalized spectra to parent material (Figure 9a and Table 3) reveal: (i) positive Ce anomalies in saprolitic, loose clayey and organo-mineral horizons with the highest values in the lower loose clayey horizon; (ii) high negative Ce anomaly in the iron duricrust horizon; (iii) slight positive Eu anomaly in the iron duricrust horizon; (iv) apart from the iron duricrust horizon ((La/Yb)_N_ = 3.92), the fractionation degree are very low along the weathering profile ((La/Yb)_N_ = 0.15–1.17) and even less than that of the parent material; (iv) the heavy REE show flat, similar and close spectra.

#### 4.2.3 Mass balance evaluation

The behaviors of elements during weathering are variable. The mass balance assessment results (losses or gains) for major and trace elements including rare-earth elements in weathered materials overlying granites are summarized in Table 5.

**Table 5:** Summary of mass balance assessment (losses and gains) for major major, trace and rare-earth elements of Biou weathering profile.

Profile zone	Sample	Losses (%, negative values)			Gains (%, positive values)		
		High >40	Moderate 40-20	Low 20-0	Low 0-100	Moderate 100-400	High >400
Coarse saprolite	BI02	Mn, U, Sr, Bi, Sm,	Na, Th, Hf, Ta, Pr, Nd, Eu, Gd, Tb,	Si, Fe, Ca, K, P, LOI, Ba, Y, Zr, Nb, Sn, La, Ce, Dy, Ho, Er, Tm, Yb, Lu	Ti, Co, Zn, Ga, Sb, W, Rb,	Mg, V, Cu, Cs	
Fine saprolite	BI03	Mn, Ca, Na, U, Sb, Cd, Sr, Nd, Sm, Eu, Gd, Tb, Dy	Y, Th, Be, Pr, Ho, Er, Tm, Yb, Lu	Si, K, P, LOI, Zr, Hf, Sn, Ta, Bi, La	Fe, Ti, Zn, Ba, Ga, Nb, Rb, Ce	Mg, Cu, Pb, Mo, W, Cs	V, Co
Lower loose cleyey horizon	BI04	Mn, Ca, Na, Zr, Be, Cd, Rb, Sr, La, Pr, Nd, Sm, Eu, Gd, Tb	Y, Sb, Dy, Ho, Er, Tm, Yb, Lu	Zn, Ba, Th, U, Sn	Si, Fe, Mg, K, Ti, P, LOI, Ga, Nb, Hf, W, Ta, Bi, Ce	Pb, Mo, Cs,	V, Co, Cu
	BI05	Mn, Ca, Na, Be, Cd, La, Pr, Nd, Sm, Eu, Gd, Tb	Sr	K, Ba, Y, Ga, Sb, Sn, Ce, Dy	Fe, Ti, P, LOI, Zn, Th, U, Z, Nb, Hf, W, Rb, Ta, Ho, Er, Tm,Yb, Lu	Si, Mg, Pb, Mo,	V, Co, Cu, Cs, Bi
	BI07	Mn, Ca, Na, Y, Be, Cd, La, Pr, Nd, Sm, Eu, Gd, Tb, Dy, Ho, Er	Ba, U, Ce, Tm, Yb, Lu	K, Zn, Th	Si, Fe, Mg, Ti, P, LOI, Ga, Zr, Sb, Nb, Hf, Sn, W, Rb, Sr, Ta	Pb, Mo, Cs, Bi	V, Co, Cu
Iron duricrust horizon	BI08	Na, K, Th, U, Zr, Nb, Hf, Cd, Sn, Ta	Ca, Ga, Be, Rb, Yb, Lu	Er, Tm	Si, LOI, Pb, Y, Sb, Bi, Ce, Tb, Dy, Ho	Ti, P, Zn, Ba, W, Sr, La, Pr, Nd, Sm, Eu, Gd,	Fe, Mn, Mg, V, Co, Cu, Mo, Cs
Upper loose clayey horizon	BI09	Mn, Ca, Ca, Be, Cd, La, Ce, Pr, Nd, Sm, Eu, Gd, Tb	Sn, Dy	Zn, Ba, Y, Ga, Un, Sb, Sr, Ho, Er, Tm, Yb, Lu	Fe, Mg, K, Ti, P, LOI, Pb, Th, Zr, Nb, Hf, W, Rb, Ta	Si, Mo, Cs	V, Co, Cu, Bi
Organo-mineral horizon	BI06	Na, Be, Cd, La, Ce, Pr, Nd, Sm, Eu, Gd	Mn, Zn, U, Sb, Sn, Tb, Dy	K, Y, Ga, Th, Ta, Ho, Er, Tm, Yb	Si, Fe, Mg, Ca, Ti, LOI, Ba, Pb, Zr, Nb, Hf, Mo, W, Rb, Sr, Bi, Lu	P, Cs	V, Co, Cu

SiO_2_, P_2_O_5_ and LOI behave similarly; they are lowly leached in saprolitic horizons and show low to moderate accumulation towards the top of the profile. P_2_O_5_ has high accumulation at the base of iron duricrust horizon (Table 5). MgO and TiO_2_ show moderate accumulation in the materials excluding the iron duricrust horizon where they have high and moderate accumulation, respectively. MnO, CaO, Na_2_O and to a lesser extent K_2_O are leached from the entire profile (Table 5). Fe_2_O_3_ is lowly leached from coarse saprolite, highly remobilized in the iron duricrust horizon and lowly accumulated in the other parts of the profile. Some trace elements (Cr, Ni, Sc and Li) with contents less than the detection limit in the parent material have significant contents in the whole weathering profile; this is the result of high remobilization during weathering (Table 5). Conversely, LILE (V, Cu, Co and Cs) which are supposed to be leached, are moderately and highly accumulated. Barium is moderately accumulated in the iron duricrust horizon. Amongst the HFSE (immobile elements: Y, Th, U, Nb, Hf, Mo, W and Ta), only Mo and W show great accumulation. In all, the iron duricrust horizon shows contrasting element distribution; for more example, Bi and Pb accumulation increases in the weathering profile from the bottom towards the top with the lowest accumulation values in the iron duricrust horizon (Table 5). REE show coherent behavior during weathering of granites in the semi-arid region of North Cameroon. In detail: (i) Ce shows low accumulation in the saprolitic horizons; (ii) Ho, Er, Tm, Yb and Lu also have low accumulation at the middle part of the lower loose clayey horizon; (iii) there is low to moderate accumulation of REE from La to Ho in the iron duricrust horizon; (iv) the depletion increases from the bottom towards the top of the weathering profile and light REE are depleted so much compared to heavy REE (Table 5).

#### 4.2.4 Enrichment factor of rare-earth elements

Mass balance evaluation and enrichment factor calculation suggest similar results of REE distribution pattern in the weathering materials underlain by S-type granites in semi-arid area (Tables 5 and 6). The results show that REE, especially light REE excluding Ce are so much enriched in the iron duricrust horizon (Table 6). Slight enrichment in Lu and Yb is registered at the base of the lower loose clayey horizon. There is Ce-enrichment in the fine saprolite (Table 6).

**Table 6:** Enrichment Factors (EF) of rare-earth elements in weathering profile from Biou area.

	Coarse saprolite	Fine saprolite	Lower loose clayey horizon			Iron duricrust horizon	Upper loose clayey horizon	Organo-mineral horizon
	BI02	BI03	BI04	BI05	BI07	BI08	BI09	BI06
La	0.82	0.83	0.24	0.19	0.38	2.88	0.14	0.31
Ce	0.91	1.09	1.23	0.81	0.68	1.00	0.33	0.59
Pr	0.73	0.67	0.21	0.17	0.37	3.10	0.14	0.31
Nd	0.70	0.65	0.20	0.18	0.36	3.17	0.15	0.30
Sm	0.60	0.44	0.20	0.20	0.33	2.72	0.20	0.32
Eu	0.61	0.49	0.25	0.31	0.36	3.03	0.25	0.47
Gd	0.65	0.48	0.29	0.37	0.32	2.28	0.35	0.54
Tb	0.77	0.58	0.46	0.59	0.39	1.61	0.52	0.72
Dy	0.85	0.65	0.61	0.81	0.45	1.31	0.65	0.79
Ho	0.83	0.73	0.79	0.97	0.49	1.19	0.80	0.85
Er	0.84	0.67	0.70	0.94	0.50	1.11	0.73	0.84
Tm	0.84	0.70	0.79	1.07	0.58	0.96	0.83	0.88
Yb	0.84	0.70	0.83	1.14	0.64	0.83	0.90	0.91
Lu	0.86	0.72	0.87	1.18	0.73	0.74	0.93	0.95

EF = [(E/Al)_sample (weathered material)_]/[(E/Al)_bacground (fresh rock)_].

## 5 Discussion

### 5.1 Petrology of granites

The bedrock sample can be classified as quartz-rich granitoids. The depletion of ferromagnesian elements (Fe, Mg, Mn, Ti, Sc, V, Cr, Co and Ni) and high contents in several trace elements (e.g., Zr, Y, Zn, Ba, Rb, Nb) are characteristic of acid rocks (Bilong et al., 2011). The high Na_2_O and K_2_O contents associated with the low MgO and CaO values could be attributed to albitization processes. The granite of Biou is S-type and peraluminous, it may derive from incongruent melting of biotite of a metasedimentary source at the bottom of the crust (Villaros et al., 2009). The albitization and greisenization processes may be responsible for high contents in several trace elements (Vriend et al., 1985). The high and variable trace element contents in these intrusive rocks may result from a mafic tendency. The high zirconium concentration, in particular, reflects coupled co-entrainment of peritectic phases and accessory minerals in the melt (Villaros et al., 2009). There are predominance and fractionation of LREE, while HREE show flat spectrum. The high contents in light REE could be due to: (i) the mantle-borne liquid enriched in light REE; (ii) the result of a low degree of partial melting; (iii) or the heavy REE preferential depletion during intrusion phase (Bilong et al., 2011). The strong Eu negative anomaly in granites may be due to plagioclase fractionation during the probably low rate partial melting (Panahi et al., 2000). Light lanthanides enrichment associated with negative Eu anomaly in the granites of Biou recalls the composition of the upper crust (e.g., Bilong et al., 2011; Silva et al., 2016).

### 5.2 Petrology of weathering materials

#### 5.2.1 Environment and weathering

**Figure 10:**
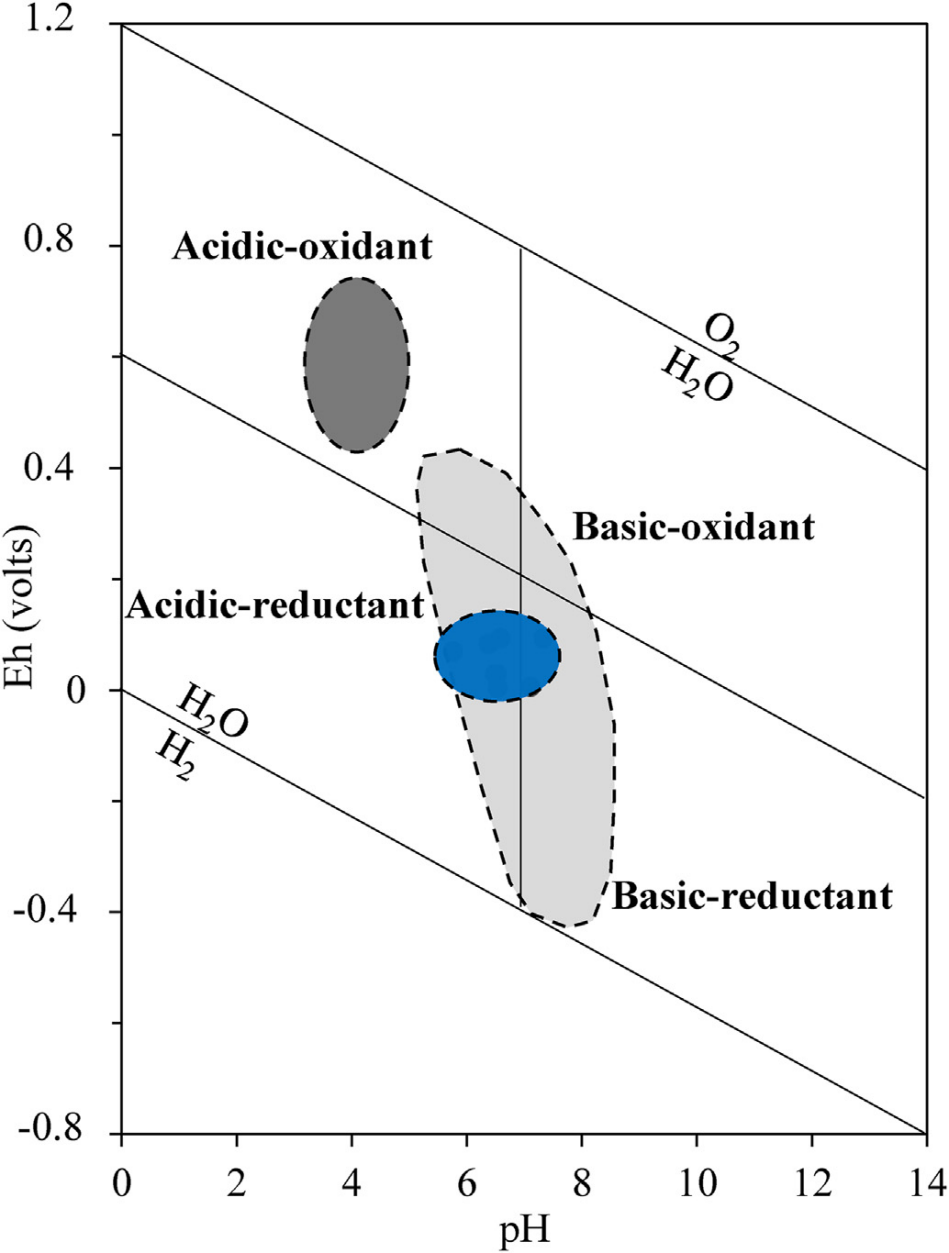
Eh-pH diagram (after Garrels and Christ, 1965) for: the Biou weathering materials (blue field), the Balkouin laterite (dark grey field) and karstic bauxite (light grey field) (Giorgis et al., 2014).

The weathering materials formation occurred at low to moderate pH and low Eh values; under acidic to basic and reducing conditions (Figure 10). These inferred conditions are similar to those of the formation of bauxite, generally obtained under reducing conditions (Giorgis et al., 2014). Conversely, the formation of Balkouin laterite have occurred under acidic and oxidizing conditions (pH ≤ 4.5 and Eh ≥ 0.4) favorable for the removal of Si and Al (Giorgis et al., 2014). In this study, the weathering materials show an increase in H_2_O and CO_2_contents with LOI values less than 1 wt% for the parent rock and rapidly pass to 8.37 wt.% in the iron duricrust horizon. The high LOI values in the whole weathering materials are a result of progressive replacement of anhydrous primary minerals by hydrous and hydroxylated minerals and hydrate oxides (Babechuk et al., 2014). Apart from iron duricrust horizon, the weathering materials have low indexes of alteration (CIA and IOL). In fact, low values of indexes of alteration indicate low weathering degree due to the slow dissolution conditions of feldspar. Not weathered granite has a CIA value between 45 and 55%, kaolinite has 100% CIA value, the values for illite varies from 75 to 90% and feldspars at 50% (Nesbitt and Young, 1982; Babechuk et al., 2014). The mineralogical composition of weathered materials (illite, muscovite, feldspar...) derived from granites in Biou area corroborates with the CIA values. The acidic and reducing conditions are favorable for the formation of illite. Under pH conditions between 5 and 7, the incongruent dissolution of feldspar preferentially leads to the neoformation of illite (Colmel-Daage and Lagache, 1965).

**Figure 11:**
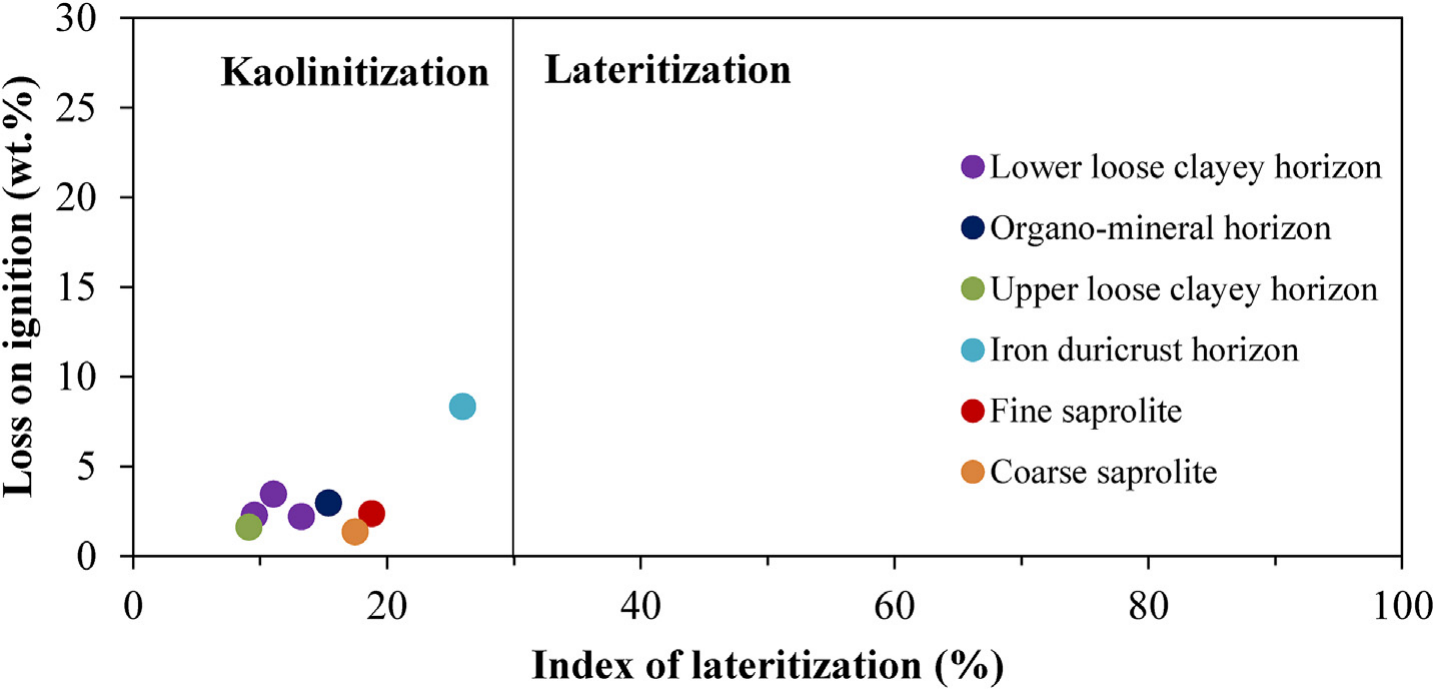
Covariation of LOI and IOL showing the process of formation of weathering materials of Biou.

**Figure 12:**
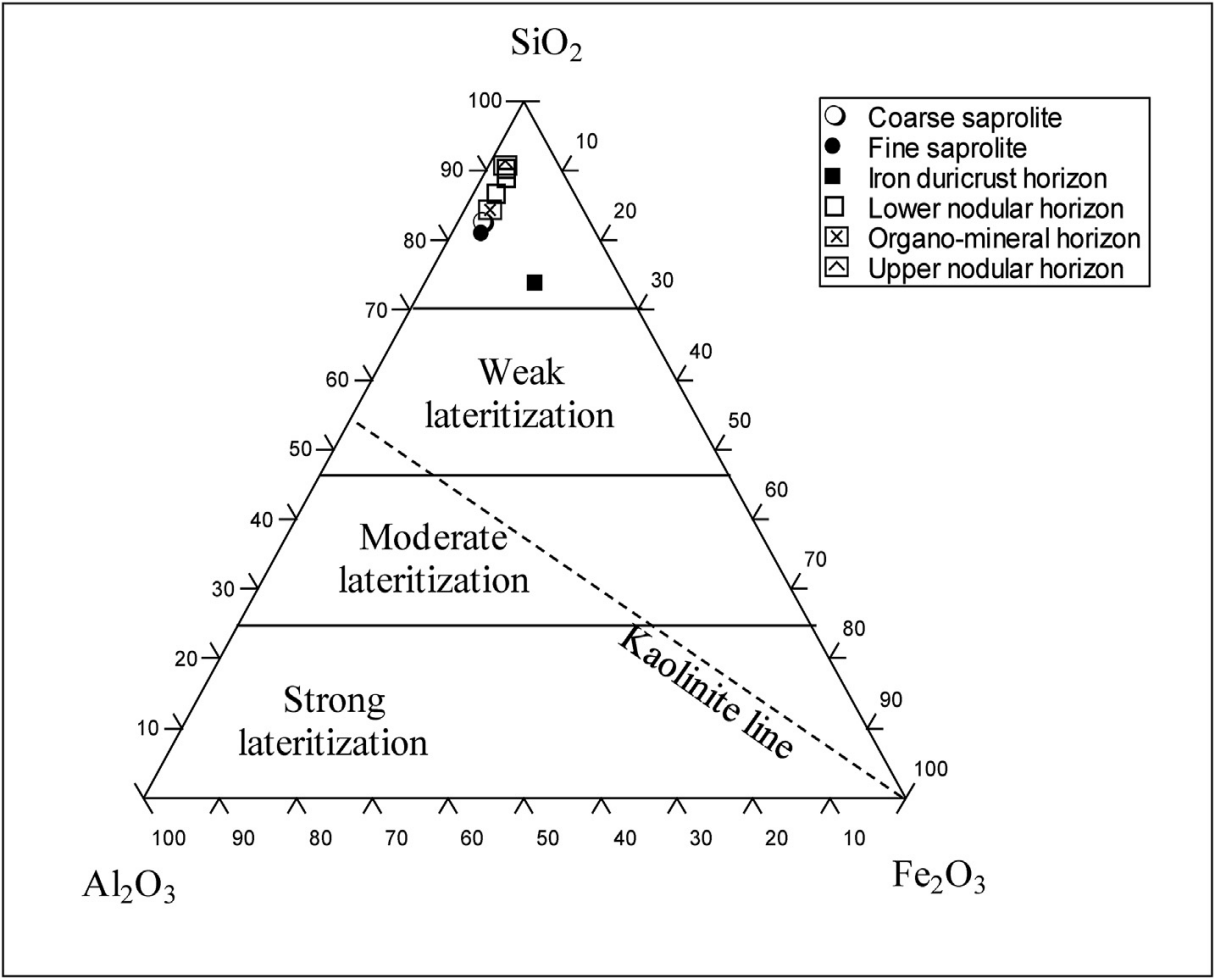
Al_2_O_3_–SiO_2_–Fe_2_O_3_ (wt.%) ternary diagram for weathering materials of Biou (after Schellmann, 1986).

Kaolinite has not been identified in the weathered samples. However, the LOI versus IOL diagram show that kaolinitization is the main process in the formation of the weathering materials developed on granites in Biou (Figure 11). According to the Schellmann (1986) classification, the samples of Biou weathering materials are outside of lateritization field (Figure 12). The iron duricrust horizon falls close to the field of lateritization (Figures 11 and 12). The Schellmann (1986) classification confirms the low proportion of secondary minerals in the whole weathering materials. The iron duricrust horizon should be considered as high weathered materials and the other horizons as moderately weathered materials.

#### 5.2.2 Chemical element distribution

The lowest accumulation of SiO_2_ in the iron duricrust horizon (Bt horizon of a Planosol) reveals the mobility of Si and the relative low SiO_2_ contents in clays. The high silica concentration in the loose clayey and organo-mineral horizons (classified as moderately weathered rock) points to the accumulation of Si at early stage of weathering. According to Eze et al. (2021), under pH higher than 8.8, Si(OH)_4_ dissociates easily as the solubility of Si compounds increases rapidly. That is why SiO_2_ accumulated in the whole weathering profile of Biou area (pH < 8.8).

The distribution of chemical elements in this weathering materials is chiefly control by the climate conditions. Ferromagnesian elements (Mg, Fe, Ti, Cr, V, Ni, Sc and Co) are generally depleted during weathering in humid and sub-humid environments due to the rapid weathering of ferromagnesian minerals. These elements are rather remobilized in weathering materials developed on S-type granites in semi-arid area of North Cameroon. The high accumulation of Ti in particular is due to the resistance of residual Ti bearing-minerals during surface processes and consequently it shows homogeneous distribution in weathering profiles (Andrade et al., 2019). The CIA values demonstrate that the weathering materials have not experienced kaolinitization processes and consequently maintain their mobile elements (Babechuk et al., 2014). This may be due to the existence of a bisiallitization process (characteristic of semi-arid climate) with the consequent formation of 2:1 secondary phyllosilicates. Potassium and sodium losses are evidenced as a consequence of chemical weathering of feldspars (Egli et al., 2001). Compared to K, Ca and Na show high losses and this is correlated to the very low resistance of plagioclase, as main source of Ca and Na, to weathering. For P behavior, Silva et al. (2020) have demonstrated that P and Mg show high accumulation in weathering materials derived from granites in dry environments.

The Al_2_O_3_–SiO_2_–Fe_2_O_3_ diagram (Figure 12) show that the samples of horizons overlying granites are aligned along the Al_2_O_3_–SiO_2_ band and progressively move towards the Fe_2_O_3_ apex for the iron duricrust horizon (the most weathered horizon). Such a behavior is highlighted by gradually accumulation of Fe oxide/hydroxide (hematite and goethite) during weathering. MgO accumulation may also be explained by the relative stability of their bearing minerals (e.g., muscovite) or the formation of illite through feldspar dissolution under acidic condition (Colmel-Daage and Lagache, 1965). The induration process in weathering profile of tropical area is always accompanied by Fe-accumulation.

The high contents of several trace elements in the weathering materials as well as their parent rock may be the result of great quantity of accessory minerals. The iron duricrust horizon constitutes a trap for most of immobile and even mobile elements. This horizon experienced REE accumulation despite their leaching in other horizons. This confirms the fact that, apart from heavy minerals, REE enrichment is found in the fine-grained samples (Cullers, 2000). Geochemical mass balance and enrichment factor data confirm the low mobility of REE, especially LREE in surface environment.

Within weathering materials, variable conditions of Eh and pH affect the mobility of chemical elements. Strong negative Ce anomaly in the iron duricrust horizon is a result of reducing conditions (Braun et al., 1990) while positive anomalies in other horizon may be due to external influence such as solutions and/or solids which possibly affected their genesis (Bourman and Ollier, 2002). The positive Eu anomaly in the iron duricrust horizon could derive from the high resistance of europium bearing-mineral under reducing conditions. The very low REE fractionation is associated to the fact that REE are not so much influenced by surface processes (Cullers and Podkovyrov, 2000).

Definitely, the geochemistry of weathering materials derived from granites in North Cameroun demonstrated the importance of the parent rock nature and the geographical setting to understand the behavior (depletion or accumulation) of elements during weathering. The very low (La/Yb)_N_ ratios and the similar distribution of REE in weathering materials are due to the environmental conditions which are not well drained (Yaboki et al., 2021).

### 5.3 REE exploration

**Figure 13:**
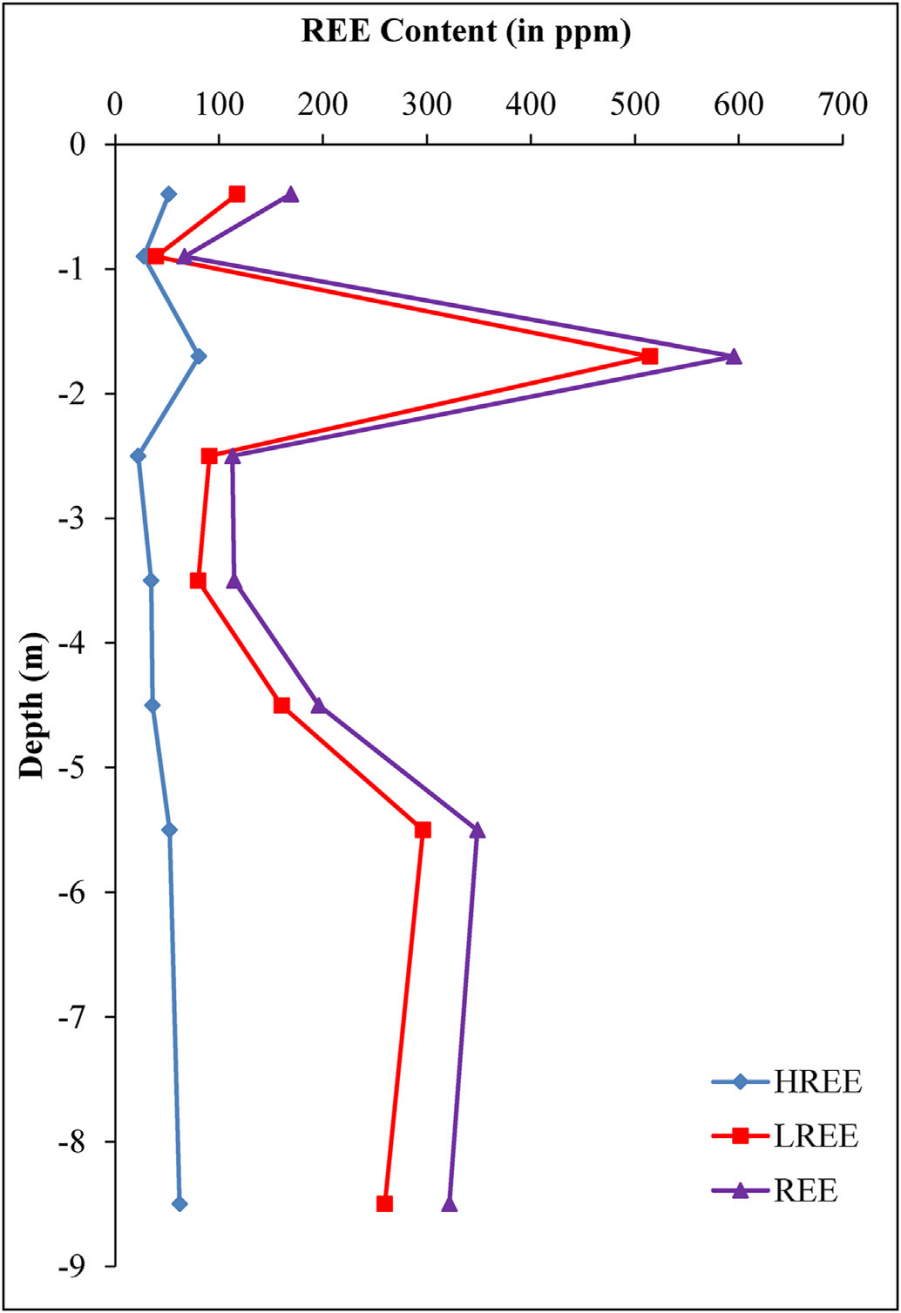
Variation of REE in the Biou weathering profile.

The significant lanthanide concentrations in the granites should be linked to the accessory minerals which are responsible for high REE contents (Saleh, 2006). Generally, monazite mostly controls LREE and zircon bears most of HREE. The presence of zircon is confirmed by the high concentration in Zr. The total REE concentration in the Biou granites is high compared to the values registered for granites from the pan-African central Cameroon by Nzenti et al. (2006) and NW Cameroon at Nyos area by Bilong et al. (2011). In addition, the data reported by Yaboki et al. (2021) on granites from Kaele (North-Cameroon) show very low REE contents. The granites of Biou seem to be favorable for further exploration as REE target at least in their weathered products. Indeed, the Pingxiang-Guangping deposits are a weathering-related REE of 10–45 m thick with contents in REE from ∼200 to 1,400 ppm in Pingxiang and from ∼150 to 1,350 ppm in Guangping (Zhang and Lin, 1996). This is due to the critical control of the primary source in the formation of REE-rich regolith. The data are similar to those of South China (Figure 8b; Fu et al., 2019). According to the variation of REE with depth diagram, the high accumulation takes place at depths 1–2.5 m (iron duricrust horizon) and 5–6 m (fine saprolite) with the peak value in the iron duricrust horizon (Figure 13). In general, REE concentrations increase from parent rock to the high weathered horizon and decrease drastically in soil (Ndjigui et al., 2013) as noticed in the Biou weathering profile. The increase in REE contents is linked to the dissolution of REE-rich primary minerals and their rapid precipitation in secondary minerals in the highly weathered horizon (Braun et al., 1990; Hoshino et al., 2016). The high REE concentrations in weathering materials could also result from the high proportion and stable nature of accessory minerals like monazite and zircon in granites (Saleh, 2006). Further investigations of the iron duricrust horizon in this area are needed for REE target. This horizon could be the ore body of REE as ion-adsorption REE deposit type after further and detail studies.

## 6 Conclusions

The high demand in REE as a result of their use in several technological industries encourages the search for new deposits. Thus, from detail characterization of weathering materials overlying granites in Biou area (region of North-Cameroon), four conclusions are draw:

- The granites are S-type, peraluminous and made up of quartz, orthoclase, microcline, plagioclase, biotite, muscovite, pyroxene and opaque minerals. They experience greisenization and albitization processes. They are rich in REE, especially light REE and show negative Eu anomalies;
- The weathering profile is well differentiated (8.5 m thick) and formed under acid and reducing conditions. Weathered fragment of rocks are abundant in the loose clayey and organo-mineral horizons. The mineralogical composition (illite, muscovite, feldspar...) and indexes of alteration indicate low weathering;
- Mass balance calculation reveals accumulation of several elements (Mg, Fe, V, Cu, Co, Cs Cr, Ni, Sc and Li) even those considered as mobile during weathering processes. Conversely, there are very low (La/Yb)_N_ ratios revealing low fractionation of REE as a result of conservative environment;
- The whole weathering materials shows high REE + Y contents just as the parent material. The high REE concentrations in the iron duricrust horizon play as the REE ore body (ion-adsorption REE deposit type).

## Declarations

### Author contribution statement

Elisé Sababa: Conceived and designed the experiments; Analyzed and interpreted the data; Wrote the paper.

Lionel G. Essomba Owona: Performed the experiments; Wrote the paper.

Jean Pierre Temga: Analysed and interpreted the data; Contributed reagents, materials, analysis tools or data.

Paul-Désiré Ndjigui: Analysed and interpreted the data; Wrote the paper.

### Funding statement

This research did not receive any specific grant from funding agencies in the public, commercial, or not-for-profit sectors.

### Data availability statement

No data was used for the research described in the article.

### Declaration of interests statement

The authors declare no conflict of interest.

### Additional information

No additional information is available for this paper.

## References

[bib1] Anderson S.P., Dietrich W.E., Brimhall G.H. (2002). Weathering profiles, mass-balance analysis, and rates of solute loss: linkages between weathering and erosion. Geol. Soc. Am. Bull..

[bib2] Andrade G.R.P., Azevedo A.C., Lepchak J.K., Assis T.C. (2019). Weathering of Permian sedimentary rocks and soil clay minerals transformations under subtropical climate, southern Brazil (Parana State). Geoderma.

[bib3] Babechuk M.G., Widdowson M., Kamber B.S. (2014). Quantifying chemical weathering intensity and trace element release from two contrasting basalt profiles, Deccan Traps, India. Chem. Geol..

[bib4] Bilong P., Ndjigui P.-D., Temdjim R., Sababa E. (2011). Geochemistry of peridotite and granite xenoliths under the early stages of weathering in the Nyos volcanic region (NWCameroon): implications for PGE exploration. Chem. Erde Geochem..

[bib5] Blaser P., Zimmermann S., Luster J., Shotyk W. (2000). Critical examination of trace element enrichments and depletions in soils: as, Cr, Cu, Ni, Pb, and Zn in Swiss forest soils. Sci. Total Environ..

[bib6] Bourman R.P., Ollier C.D. (2002). A critique of the Schellmann definition and classification of ‘laterite. Catena.

[bib7] Brabant P., Gavaud M. (1995).

[bib8] Braun J.J., Pagel M., Muller J.P., Bilong P., Michard A., Guillet B. (1990). Cerium anomalies in lateritic profiles. Geochem. Cosmochim. Acta.

[bib9] Braun J.-J., Viers J., Dupre B., Polve M., Ndam J., Muller J.-P. (1998). Solid/liquid REE fractionation in the lateritic system of Goyoum, East Cameroon: the implication for the present dynamics of the soil covers of the humid tropical regions. Geochem. Cosmochim. Acta.

[bib10] Burnham O.M., Schweyer J. (2004). Trace element analysis of geological samples by inductively coupled plasma-mass spectrometry at the Geoscience Laboratories: revised capacities due to improvements to instrumentation. Ontario Geol. Surv..

[bib11] Cao X., Wu P., Cao Z. (2016). Element geochemical characteristics of a soil profile developed on dolostone in central Guizhou, southern China: implications for parent materials. Acta Geochim..

[bib12] Castor S.B. (2008). The Mountain Pass rare-earth carbonatite and associated ultrapotassic rocks, California. Can. Mineral..

[bib13] Chappell B.W., White A.J.R. (1974). Two contrasting granite types. Pac. Geol..

[bib14] Chu H., Chi G., Bosman S., Card C. (2015). Diagenetic and geochemical studies of sandstones from drill core DV10-001 in the Athabasca basin, Canada, and implications for uranium mineralization. J. Geochem. Explor..

[bib15] Colmel-Daage E., Lagache P. (1965). Caractéristiques de quelques groupes de sols dérivés de roches volcaniques aux Antilles françaises. Cah. O.R.S.T.O.M. Pedol..

[bib16] Cullers R.L. (2000). The geochemistry of shales, siltstones and sandstones of Pennsylvanian Permian age, Colorado, U.S.A.: implications for provenance and metamorphic studies. Lithos.

[bib17] Cullers R.L., Podkovyrov V.N. (2000). Geochemistry of the Mesoproterozoic Lakhanda shales in southeastern Yakutia, Russia: implications for mineralogical and provenance control, and recycling. Precambrian Res..

[bib18] Da Silva Y.J.A.B., Do Nascimento C.W.A., Biondi C.M. (2017). Influence of metaluminous granite mineralogy on the rare earth element geochemistry of rocks and soils along a climosequence in Brazil. Geoderma.

[bib19] Ebah Abeng S.A., Ndjigui P.-D., Aye A.B., Tessontsap T., Bilong P. (2012). Geochemistry of pyroxenites, amphibolites and their weathered products in the Nyong unit, SW Cameroon (NW border of Congo craton): implications for Au–PGE exploration. J. Geochem. Explor..

[bib20] Egli M., Mirabella A., Fitze P. (2001). Weathering and evolution of soils formed on granitic, glacial deposits: results from chronosequences of Swiss alpine environments. Catena.

[bib21] Eze P.N., Molwalefhe L.N., Kebonye N.M. (2021). Geochemistry of soils of a deep pedon in the Okavango Delta, NW Botswana: Implications for pedogenesis in semi-arid regions. Geoderma Reg..

[bib22] Fan H.-R., Yang K.-F., Hu F.-F., Liu S., Wang K.-Y. (2016). The giant Bayan Obo REE-Nb-Fe deposit, China: controversy and ore genesis. Geosci. Front. Times.

[bib23] Fu W., Li X., Feng Y., Feng M., Peng Z., Yu H., Lin H. (2019). Chemical weathering of Stype granite and formation of Rare Earth Element (REE)-rich regolith in South China: Critical control of lithology. Chem. Geol..

[bib24] Gong Q., Deng J., Wang C., Wang Z., Zhou L. (2013). Element behaviors due to rock weathering and its implication to geochemical anomaly recognition: a case study on Linglong biotite granite in Jiaodong peninsula, China. J. Geochem. Explor..

[bib25] Gong Q., Deng J., Jia Y., Tong Y., Liu N. (2015). Empirical equations to describe trace element behaviors due to rock weathering in China. J. Geochem. Explor..

[bib75] Garrels R.M., Christ C.L. (1965). Solutions, Minerals, and Equilibria.

[bib26] Giorgis I., Bonetto S., Giustetto R., Lawane A., Pantet A., Rossetti P., Thomassin J.-H., Vinai R. (2014). The lateritic profile of Balkouin, Burkina Faso: Geochemistry, mineralogy and genesis. J. Afr. Earth Sci..

[bib27] Grant J.A. (2005). Isocon analysis: a brief review of the method and applications. Phys. Chem. Earth.

[bib28] Guiraud R., Bellion Y., Benkhelil J., Moreau C. (1987). Post-Hercynian Tectonics in Northern and Western Africa. Geol. J..

[bib74] Hoshino M., Sanematsu K., Watanabe Y. (2016).

[bib29] Hu Y., Liu X., Bai J., Shih K., Zeng E.Y., Cheng H.H. (2013). Assessing heavy metal pollution in the surface soils of a region that had undergone three decades of intense industrialization and urbanization. Environ. Sci. Pollut. Res..

[bib30] Islam M.R., Peuraniemi V., Aario R., Rojstaczer S. (2002). Geochemistry and mineralogy of saprolite in Finnish Lapland. Appl. Geochem..

[bib31] Jiménez-Espinosa R., Vázquez M., Jiménez-Millán J. (2007). Differential weathering of granitic stocks and landscape effects in a Mediterranean climate, Southern Iberian Massif (Spain). Catena.

[bib32] Kessoum Adamou J.-M., Noa Tang S.D., Sababa E., Onana V.L. (2021). Weathering profiles developed on gneisses from Batchenga and Doua areas, central Cameroon: Climate and topography controls. J. Afr. Earth Sci..

[bib33] Köppen W. (1918). Klassification der Klimate nach Temperatur, Niederschlag and Jahreslauf. Petermanns Geogr. Mittl..

[bib34] Letouzey R. (1985). Carte phytog_eographique du Cameroun au 1/50000 avec notice explicative. Inst. la carte Int. la v_eg. Fasc.

[bib35] Liu Y., Hou Z. (2017). A synthesis of mineralization styles with an integrated genetic model of carbonatite-syenite-hosted REE deposits in the Cenozoic Mianning-Dechang REE metallogenic belt, the eastern Tibetan Plateau, southwestern China. J. Afr. Earth Sci..

[bib36] Li Y.H.M., Zhao W.W., Zhou M.-F. (2017). Nature of parent rocks, mineralization styles and ore genesis of regolith-hosted REE deposits in South China: an integrated geneticmodel. J. Afr. Earth Sci..

[bib37] Liu R., Wang R.C., Lu X., Li J. (2016). Nano-sized rare earth minerals from granite-related weathering-type REE deposits in southern Jiangxi. Acta Petrol. Mineral..

[bib38] McLennan S.M. (1993). Weathering and global denudation. J. Geol..

[bib39] Miao L., Xu R., Xu J. (2007). Geochemical characteristics of rare earth elements (REEs) in the soil-plant system in West Guangdong Province. Acta Pedol. Sin..

[bib40] Mihajlovic J., Rinklebe J. (2018). Rare earth elements in German soils - A review. Chemosphere.

[bib41] Ndjigui P.-D., Badinane M.F.B., Nyeck B., Nandjip H.P.K., Bilong P. (2013). Mineralogical and geochemical features of the coarse saprolite developed on orthogeneiss in the SW of Yaounde, South Cameroon. J. Afr. Earth Sci..

[bib42] Nesbitt H.W., Markovics G. (1997). Weathering of granodioritic crust, long term storage of elements in weathering profils, and petrogenesis of siliciclastic sediments. Geochem. Cosmochim. Acta.

[bib43] Nesbitt H.W., Young G.M. (1982). Early Proterozoic climates and plate motions inferred from major element chemistry of lutites. Nature.

[bib44] Nyassa Ohandja H., Onana V.L., Noa Tang S.D., Ngo’o Ze A., Ekodeck G.E. (2020). Behavior of major, trace, and rare earth elements in an atypical lateritic profile overlying micaceous quartzites, Centre Cameroon: imprint of the parent rock structure. Arab. J. Geosci..

[bib45] Nzenti J.-P., Kapajika B., Wörner G., Lubala T.R. (2006). Synkinematic emplacement of granitoids in a Pan-African shear zone in Central Cameroon. J. Afr. Earth Sci..

[bib72] Panahi A., Young G.M., Rainbird R.H. (2000). Behavior of major and trace elements (including REE) during Paleoproterozoic pedogenesis and diagenetic alteration of an Archean granite near Ville Marie, Québec, Canada. Geochem. Cosmochim. Acta.

[bib46] Penaye J., Kröner A., Toteu S.F., Van Schmus W.R., Doumnang J.C. (2006). Evolution of the Mayo-Kebbi region as revealed by zircon dating: an early (ca. 740 Ma) Pan-African magmatic arc in southwestern Chad. J. Afr. Earth Sci..

[bib47] Pourmand A., Dauphas N., Ireland T.J. (2012). A novel extraction chromatography and MC-ICP-MS technique for rapid analysis of REE, Sc and Y: Revising CI-chondrite and Post-Archean Australian Shale (PAAS) abundances. Chem. Geol..

[bib48] Roskill (2011).

[bib49] Sababa E., Fuh Calistus G., Ndjigui P.-D., Onana P.N., Djimet Tetedjima S. (2021). Petrography and geochemistry of sulfurous volcanic scoria from mount Cameroon area, Central Africa: Implications for Au-PGE exploration. J. Afr. Earth Sci..

[bib50] Sababa E., Ndjigui P.-D., Ebah Abeng S.A., Bilong P. (2015). Geochemistry of peridotite xenoliths from the Kumba and Nyos areas (southern part of the Cameroon Volcanic Line): implications for Au-PGE exploration. J. Geochem. Explor..

[bib51] Saleh G.M. (2006). Uranium mineralization in the muscovite-rich granites of the Shalatin region, Southeastern Desert, Egypt. Chin. J. Geochem..

[bib52] Sanematsu K., Kon Y., Imai A., Watanabe K., Watanabe Y. (2013). Geochemical and mineralogical characteristics of ion-adsorption type REE mineralization in Phuket, Thailand. Miner. Deposita.

[bib53] Sanematsu K., Watanabe Y. (2016). Characteristics and genesis of ion-adsorption type deposits. Econ. Geol..

[bib54] Schellmann W. (1986). A new definition of laterite. Geol. Surv. India Memoir..

[bib55] Silva C.M.C.A.C., Nascimento R.C., da Silva Y.J.A.B., Barbosa R.S., da Silva Y.J.A.B., do Nascimento C.W.A., van Straaten P. (2020). Combining geospatial analyses to optimize quality reference values of rare earth elements in soils. Environ. Monit. Assess..

[bib56] Silva M.V.M.G., Pinto M.M.S.C., Carvalho P.C.S. (2016). Major, trace and REE geochemistry of recent sediments from lower Catumbela River (Angola). J. Afr. Earth Sci..

[bib57] Silva Y.J.A.B., Nascimento C.W.A., Van Straaten P., Biondi C.M., Silva Y.J.A.B., Araujo J.C.T., Alcantara V.C., Silva F.L., Silva R.J.A.B. (2018). Rare earth element geochemistry during weathering of S-type granites from dry to humid climates of Brazil. J. Plant Nutr. Soil Sci..

[bib58] Smith M., Moore K., Kavecsánszki D., Finch A.A., Kynicky J., Wall F. (2016). From mantle to critical zone: A review of large and giant sized deposits of the rare earth elements. Geosci. Front..

[bib59] Suchel J.B. (1987).

[bib60] Temga J.P., Sababa E., Mamdem L.E., Ngo Bidjeck M.L., Tamfuh Azinwi P., Tehna N., Zo’o Zame P., Onana V.L., Nguetnkam J.P., Bitom L.D., Ndjigui P.-D. (2021). Rare earth elements in tropical soils, Cameroon soils (Central Africa). Geoderma Reg..

[bib61] Toteu S.F., Penaye J., Deloule E., Van Schmus W.R., Tchameni R. (2006). Diachronous evolution of volcano-sedimentary basins north of the Congo craton: insights from U–Pb ion microprobe dating of zircons from the Poli, Lom and Yaounde Series (Cameroon). J. Afr. Earth Sci..

[bib62] Toteu S.F., Penaye J., Poudjom Djomani Y. (2004). Geodynamic evolution of the Pan-African belt of Central Africa with special reference to Cameroon. Can. J. Earth Sci..

[bib63] Ufer K., Stanjek H., Roth G., Dohrmann R., Kleeberg R., Kaufhold S. (2008). Quantitative phase analysis of bentonites by the Rietveld method. Clay Miner..

[bib65] Verplanck P., Mariano A., Mariano A. (2016). Rare earth element ore geology of carbonatites. Econ. Geol..

[bib66] Villaros A., Stevens G., Moyen J.-F., Buick I.S. (2009). The trace element compositions of S-type granites: evidence for the disequilibrium melting and accessory phase entrainment in the source. Contrib. Mineral. Petrol..

[bib67] Vriend S.P., Oosteron M.G., Bussink R.W., Jansen J.B.H. (1985). Trace element behavior in the W-Sn granite of Regoufe, Portugal. J. Geochem. Explor..

[bib69] Yaboki E., Temga J.P., Sababa E., Djakba Basga S., Nguetnkam J.P. (2021). Geochemical Characterization of Vertisols Developed on Granites from Kaele, North-Cameroon: Implications for REE Exploration. WJAS.

[bib70] Yang X.J., Lin A., Li X.-L., Wu Y., Zhou W., Chen Z. (2013). China's ion-adsorption rare earth resources, mining consequences and preservation. Environ. Dev..

[bib71] Zhang Z., Lin C. (1996). The behaviour of rare-earth elements (REE) during weathering of granites in southern Guangxi, China. Chin. J. Geochem..

